# Vegan Diets for Children: A Narrative Review of Position Papers Published by Relevant Associations

**DOI:** 10.3390/nu15224715

**Published:** 2023-11-07

**Authors:** Boštjan Jakše, Zlatko Fras, Nataša Fidler Mis

**Affiliations:** 1 Independent Researcher, 1000 Ljubljana, Slovenia; bj7899@student.uni-lj.si; 2 Division of Medicine, Centre for Preventive Cardiology, University Medical Centre Ljubljana, 1000 Ljubljana, Slovenia; zlatko.fras@kclj.si; 3 Faculty of Medicine, University of Ljubljana, 1000 Ljubljana, Slovenia; 4 Ministry of Health, 1000 Ljubljana, Slovenia; 5 Division of Paediatrics, University Medical Centre Ljubljana, 1000 Ljubljana, Slovenia

**Keywords:** vegan diet, children, adolescents, position statement, expert opinion

## Abstract

The scientific discourse on vegan diets for children and adolescents primarily involves referencing position statement papers from different scientific and professional organizations, including paediatric associations. Over the past two decades, specialized associations have issued official statements and published position papers about adopting well-designed vegan diets during crucial life stages, including pregnancy and lactation, infancy, and childhood. A subset of these associations firmly supports the notion that a well-designed vegan diet can indeed be healthy and support normal growth and development during particularly delicate life stages, emphasizing careful planning, vitamin B12 supplementation, and regular supervised medical and dietetics oversight. In contrast, specific paediatric associations caution against vegan diets for children and adolescents, citing potential harm and the lack of adequate substantiation. These criticisms in position papers frequently point to lower-quality studies and/or outdated studies. Additionally, concerns extend to comparing vegan and omnivorous diets, considering public health issues such as obesity and early stages of cardiovascular disease as well as the risk of prediabetes and type 2 diabetes. Notably, some scepticism stems from studies where children’s adherence to a well-designed vegan diet is incomplete. Scientific rigor suggests performing a comparable assessment of omnivorous and vegan diets. This narrative review highlights the need for a comprehensive, up-to-date literature review to inform balanced perspectives on vegan diets for children and adolescents. Researchers and decision-makers should aim to actively improve the design and consistent implementation of both diet types.

## 1. Introduction

Nutrition during the perinatal period and early development has a significant impact on long-term health into adulthood (i.e., food (nutrition) preferences, adult body mass, and common chronic diseases) [1,2,3]. The escalating prevalence of overweight and obesity in children and adolescents has evolved into a significant global epidemic, contributing to substantial societal and health care challenges worldwide [4,5]. Such a situation is particularly concerning given that obesity is one of the primary contributing factors to the development of cardiovascular diseases (CVDs) [6]. Coronary atherosclerosis starts to develop prenatally. Fatty streak formation occurs in human foetal aortas and is highly enhanced by maternal hypercholesterolemia. The aortas of foetuses from mothers with hypercholesterolaemia and temporary hypercholesterolemia contain significantly larger lesions than those of foetuses from mothers with normocholesterolaemia [7,8,9,10]. Intimal accumulation and oxidation of low-density lipoprotein cholesterol precede monocyte recruitment into early atherosclerotic lesions [11]. Additionally, high birth body mass is associated with obesity and increased intima-media thickness of the carotid arteries in young adults. Young adults born large for gestational age are more likely to become obese and to show increased carotid intima-media thickness, a marker of subclinical atherosclerosis, consistent with an increased risk of CVD [12]. Decades ago, autopsies of adolescents and young adults who had died from various (mainly external) causes revealed that coronary atherosclerosis is initiated in youth [9,10,11,12]. An investigation utilized cardiac magnetic resonance imaging to compare overweight and obese children (average age of 12 years) with normal-weight children (average age of 14 years) and found that overweight and obese children exhibited significant increases in the size of the left ventricle and substantial thickening of the heart muscle, indicating concentric heart hypertrophy [6].

Over the past two decades, there has been a growing trend of families choosing to consume vegan (VN) diets. In adults, the consumption of a VN diet is associated with a decreased risk of various common chronic noncommunicable diseases (NCDs), especially obesity, CVD, type 2 diabetes, and some cancers [13,14,15,16]. Furthermore, VN diets are also recognized as much more environmentally sustainable than omnivorous (OM) diets [17,18,19]. The appropriateness of a VN diet can be assessed through various criteria, such as the impact on general health, nutritional adequacy, and the growth and development of children, as well as the incidence of paediatric diseases, the prevalence of obesity and other common chronic diseases in adulthood, health care costs, food costs, environmental sustainability, and ethical concerns. Limitations in meeting these criteria include a scarcity of well-designed studies on children consuming VN as well as OM diets, especially randomized clinical trials and long-term prospective studies. Indeed, this shortage of high-quality, well-designed studies can be attributed to difficulties in recruiting parents of VN children, often due to concerns about potential criticism from the medical community [20,21].

Of importance, a well-designed VN diet is a plant-based eating pattern planned and executed in a healthy and appropriate manner, meaning it is energetically and nutritionally sufficient to meet nutritional recommendations. Furthermore, it involves carefully selecting and preparing a variety of nutrient-rich, whole-plant foods, such as fruits, vegetables, whole grains, legumes, nuts, and seeds, and limiting the intake of highly processed foods that are rich in sugar, salt, and preservatives, ensuring that all daily nutritional needs are met. Practising a well-designed VN diet includes paying attention to portion sizes, food combinations, and the balance of nutrients to ensure optimal health and well-being. Finally, it is necessary to add vitamin B12, and potentially also EPA/DHA and vitamin D to the diet if an individual lives at a latitude above 40° during wintertime (October to April). During this period, vitamin D supplementation is essential, as sun exposure alone does not produce enough vitamin D. This is universal to all dietary patterns [22,23]. A recent narrative review including 360 families in Italy revealed that nearly half of the surveyed parents expressed dissatisfaction with their paediatricians’ preparedness to offer comprehensive guidance and suitable recommendations for nontraditional weaning dietary practices, especially for vegetarian (VG) and VN diets. Moreover, a noteworthy 77% of parents reported facing paediatrician resistance towards these alternative weaning methods. Recently, it was reported that the breastfeeding duration was 6 months longer in infants consuming VG/VN diets than in infants consuming OM diets (15.8 months vs. 9.7 months) [24]. Apprehension regarding the inadequacy of certain nutrients within a VN diet, particularly during early life stages, carries substantial significance. Nevertheless, society would have to confront more pressing challenges stemming from the consequences of the obesogenic food milieu. Such an environment exacerbates the prevailing public health burdens, including the burdens of obesity, type 2 diabetes, and increasingly widespread chronic NCDs. These issues are fuelled by a nutritional pattern characterized by food addiction and overconsumption, featuring an overabundance of energy-dense foods, free sugars, especially in liquid form (i.e., sugar-sweetened beverages), highly processed carbohydrates, and trans, saturated, and total fats, as well as cholesterol [25,26,27]. Regardless, the suitability of VN diets for children and adolescents remains a subject of ongoing research and debate.

A recent review study analysed the existing scientific literature on the health effects of VN diets on children’s growth. The authors concluded that, with a small percentage of outliers, VN children had normal growth and were less often obese. They found limited evidence that children consuming VN diets could obtain all the examined nutrients. As proper planning and supplementation by caregivers is needed, it is currently yet to be determined how often VN children follow well-planned diets. Deficiencies in vitamin B_12_, calcium, and vitamin D are the most significant risks associated with a poorly planned VN diet. Data on the dietary intake and nutrient status of omega-3 fatty acids, zinc, iodine, and selenium are still needed. Future research should discriminate between VN subpopulations that are open or closed towards the scientific approach, general health questions, and supplementation. There is a need for studies assessing the modes of supplementation and supplement dosages, the use of fortified foods or drinks, and adherence to the VN diet itself. Most studies encompassed in the review date back over 15 to 40 years. Taking into account both adult-oriented research on VN diets and the methodological nuances inherent in the analysed studies focused on children’s diets (i.e., different methods for the estimation of dietary intake, limitations of each of the methods, incomplete food composition databases of foods, and limited data for nutrients in the studies), the review concluded that a well-designed and supplemented VN diet is likely to support optimal child health and development [28]. In a recent study of women raising their toddlers on various types of VG and OM diets, those who raised their toddlers on VN diets demonstrated a higher awareness of the potential for nutritional deficiencies and administered dietary supplements more frequently than those who raised their children on OM diets. The authors also stressed that a VG diet could be safe for young children. However, parents should be educated about the risk of nutritional deficiencies and the principles of healthy nutrition regardless of the administered diet, and effective communication among parents, paediatricians, and dietitians should be the cornerstone of every nutritional strategy in managing VG children [29].

Critics who question the suitability of VN diets for children often emphasize a specific point in their discussions. They typically underline that the positive health benefits observed in adults may not necessarily apply directly to children. Interestingly, these critics primarily assess and evaluate potential risks associated with VN diets, often relying on studies involving adults as their basis. A common criticism of the suitability of VN diets for children revolves around the potential for reduced bone mineral density and a potentially elevated susceptibility to bone fractures. When challenging the viability of VN diets for children, authors sometimes overemphasize the obtained differences that are not clinically relevant and selectively reference less favourable data from adults following VN diets. For instance, a recent German study comparing bone health in VN (*n* = 36) and OM (*n* = 36) adults found a 5% difference in one bone health measurement. However, the clinical relevance of this small study is limited due to the lack of adjustment for BMI, while the trends suggest potential bone health improvement with longer VN diet adherence [30]. Furthermore, a UK study from Epic-Oxford (initial data collected in 1993–2001, with follow-up performed in approximately 2010) revealed higher fracture rates among VN individuals, even after adjusting for BMI, calcium intake, and bone density. With a high degree of certainty, this was attributed to poorly designed diets with high fat intake (28% of energy) and low fibre (26 g/day), calcium (600 mg/day), vitamin D (assumed due to the high geographical latitude) [31], and possibly vitamin B_12_ [32] intake, along with insufficient physical activity levels [33]. This interpretation aligns with a recent UK prospective cohort study (initial data collected in 2006–2010, with follow-up in 2021), indicating that VG individuals had a higher risk of hip fracture than regular OM individuals, partly explained by their lower BMIs. Notably, this study involved an overweight VG population with even lower postpeak physical activity levels than the OM population. The VG group consumed sugary drinks at 31 times higher amounts than the comparison group (47 mL/day vs. 1.5 mL/day) and had notably low fibre intake (23 g/day), high total fat intake (36% of energy), similar saturated fatty acid (SFA) intake, and deficiencies in essential nutrients such as vitamin C, vitamin D, folate, potassium, iron, and zinc [34].

It is well known that meta-analyses can sometimes result in nonobjective interpretations [35]. For instance, a recent systematic review and meta-analysis of 17 cross-sectional studies from Chinese researchers exploring the link between VG diets (including VN diets) and OM diets and bone health faced limitations due to the inclusion of only five VN-focused studies. Among these studies, one involved individuals following raw VN diets, another focused on Buddhist nuns, and a third centred around an older adult population (aged 70–89 years). It is important to note that, in one of the studies, the protein intake of VN subjects was merely 35 g/day. In contrast, two studies demonstrated that VN subjects had comparable bone mineral density to OM subjects [36]. These sources will undergo deeper scrutiny in forthcoming chapters of the present paper.

This narrative review consisted of two parts. First, we aimed to include scientific/professional associations and expert group position statements or opinions regarding the suitability of VN diets for children and adolescents available in PubMed/Medline or published on the websites of the institutions of these expert groups. In addition, we searched PubMed/Medline and web pages for relevant position statements and expert group position statements or opinions on this topic for Slovenia, as these significantly impact the dietary guidelines and nutrition practices for the paediatric population in Slovenia. Second, we reviewed the primary studies involving children and adolescents who consumed VG diets (including VN diets) and OM diets published in the PubMed/Medline, Scopus, and Web of Science databases from April 2015 to October 2023. These studies should be included in upcoming position papers. The many newer studies would be of interest to the relevant associations taking positions on the suitability of VN diets for children and adolescents. Our main objectives were (1) to evaluate the scientific rigor of the position papers; (2) to explore recent studies comparing VN and OM diets in children and adolescents (most of them were not included within past position papers); and (3) to investigate whether VN diets for children are less healthy, comparably healthy, or healthier than OM diets. We considered position papers and official documents addressing clinical issues [37] as part of the first objective. We offer consensus-based solutions to provide a unified perspective in areas with differing practices. For the second objective, we employed a method in which each of the key study characteristics is presented comprehensively in the narrative review, along with concise comments. Such an approach aims to enhance the reader’s comprehensive understanding by presenting essential consolidated insights. Concerning the third objective, most comparative studies on VN diet outcomes left the implications of OM diets largely unexplored.

## 2. Positions of the Relevant Associations Regarding Vegan Diets for Children

Over the last twenty years, many scientific and professional associations from the field of nutrition and paediatrics have published position statement papers on the suitability of VN diets for children and adolescents [38,39,40,41,42,43,44,45,46]. Table 1 presents different scientific and professional associations and expert groups that support or are reserved/dismissive regarding VG and VN diets during all stages of life, including pregnancy and lactation, for infants, children, adolescents, adults, and elderly individuals, as well as athletes. The majority of them hold the position that a well-designed VN diet supplemented with vitamin B12 is healthy and appropriate during these susceptible life periods. However, all of the associations and groups clearly emphasize that a VN diet requires more careful planning and appropriate supplementation [21,42,43].

Conversely, German and French paediatricians are more reserved [47] or dismissive [48] regarding the suitability of VN diets for children and adolescents. The German standpoint adopts a more prudent approach, emphasizing heightened vigilance towards the intake and levels of specific nutrients such as iron, zinc, iodine, docosahexaenoic acid (DHA), calcium, and protein and energy intake. This is aimed at preventing critical clinical consequences, including growth impediments, anaemia, or neurological impairments [47]. In contrast, the stance adopted by the French paediatric profession is a nonrecommendation of VN diets for infants, children, and adolescents. Such a perspective is founded on the perceived inevitability of nutritional deficiencies arising from the absence or lack of appropriate supplements. In addition, it suggests that the suitability of VN diets for humans is questionable [48]. Nevertheless, the German perspective is founded on the earlier 2016 edition and a citation dating back three and a half decades [47]. In contrast, the French stance is mostly derived from the analysis of two cross-sectional studies [48]. A decade-old study centred around a family that followed a VN diet, encompassing parents and two children (a 10-month-old girl and a 12-month-old boy), who presumably, according to the authors, suffered from anaemia and delayed development due to vitamin B12 deficiency caused by the mother following a strict VN diet during pregnancy and lactation [49]. Concurrently, another 20-year-old study investigated a group of 30 young individuals (*n* = 15 males and *n* = 15 females) aged 16 to 20 years who had adhered to VN diets for 1.7 years. This study involved a comparison with 30 age-matched individuals practising OM diets. The most significant findings (excluding supplement intake) were that VN individuals had higher fibre intake (males: 44 g/day vs. 25 g/day, females: 34 g/day vs. 21 g/day), lower SFA intake (males: 8% of energy intake vs. 13% of energy intake, females: 6% of energy intake vs. 13% of energy intake), and less protein intake (males: 10% of energy intake vs. 15% of energy intake, females: 10% of energy intake vs. 14% of energy intake) than OM individuals. Regarding micronutrients, VN individuals had no vitamin B12 intake (while the OM individuals had sufficient intake), insufficient calcium and selenium intake, and sufficient iron and zinc intake. Insufficient iron status was higher among OM females (23%) than among VN females (20%), indicating a female sex rather than a VN diet problem. Iron deficiency anaemia was more common among OM individuals than VN individuals (20% vs. 7%). Additionally, VN males had lower body mass and BMI than OM males [50]. In 2019, the Royal Academy of Medicine of Belgium, an advisory agency for Belgium’s government institutions, released a brief and poorly referenced publication suggesting that the consumption of VN diets poses risks to pregnant women and children. The committee considers VN diets to be unsuitable and does not recommend them for unborn children, children and adolescents, and pregnant and breastfeeding women [51]. Furthermore, this position faced significant criticism from many nutrition experts (health professional organizations), who viewed it as an attempt to criminalize parents who follow VN diets for their children [52,53,54]. Despite these concerns about VN diets, Belgium has broader dietary challenges. Only 3% of young Flemish adults consume the recommended amount of vegetables. Belgians across all age groups also fall short on fibre intake, an essential nutrient found only in plants. Flemish millennials, but not VG individuals, on average, consume less than 150 g of the recommended 300 g of vegetables daily [55,56,57].

Numerous experts and individuals commonly allude to the favourable stance regarding the suitability of VN diets for children articulated by the American Academy of Nutrition and Dietetics (AND) in 1997 [58], 2003 [46], 2009 [45], and 2016 [43]. These positions of the AND are among the earliest to endorse a well-designed VN diet throughout various life stages. They draw upon a comprehensive body of literature to support their viewpoints, primarily relying on direct evidence from various studies involving adults who followed VN diets. Similarly, Canadian dietitians, in alignment with the American Dietetic Association at the time, asserted twenty years ago that a well-designed VN diet can fulfil current nutrient requirements and is suitable for all life phases, encompassing pregnancy, breastfeeding, infancy, childhood, adolescence, and adulthood [46]. Furthermore, in 2010, upon revisiting the data, they reaffirmed their position and introduced some additional recommendations. These recommendations included emphasizing adequate energy intake, the intake of specific nutrients, and the necessity of supplementing with at least vitamin B12. Their ultimate finding asserts that ample evidence supports the notion that children and adolescents can achieve healthy growth and overall well-being through a well-designed VN diet that includes appropriate supplementation. Additionally, they highlighted routine vitamin D supplementation for infants, irrespective of their dietary pattern [42]. Fifteen years ago, the American Academy of Pediatrics endorsed a well-designed VN diet as a healthy alternative lifestyle at all stages of foetal, infant, child, and adolescent growth [59]. It is noteworthy to include the stance articulated by the North American Society for Pediatric Gastroenterology, Hepatology, and Nutrition (NASPGHAN) concerning VN formulas. This perspective is particularly relevant when breastfeeding is not feasible due to adherence to a dairy-free diet during the first year of life. The NASPGHAN emphasizes the significance of appropriately formulated VN formulas, such as those derived from soy or rice, which can serve as a viable substitute for cow milk-based formulas [60].

In Europe, many experts often rely on the position of the European Society for Paediatric Gastroenterology, Hepatology, and Nutrition (ESPGHAN). The ESPGHAN position paper considers different aspects of complementary feeding, focusing on healthy term infants in Europe (age 4–12 months). Concerning VN diets for infants, the authors concluded that “VN diets with appropriate supplementation can support normal growth and development. Regular medical and dietetic supervision should be given and followed to ensure the nutritional adequacy of the diet. The consequences of failing to do this can be severe and include irreversible cognitive impairment and death” [3]. In the methodology section, the term vegan nutrition was not cited under search terms, and the authors only cited one reference on VG nutrition in infants and children published over ten years ago. In their position paper on the role of dietary factors and food habits in the development of childhood obesity, the ESPGHAN states that “Plant foods can be used as the main food contributors to a well-balanced diet. When a VG diet is practised, appropriate planning (taking into account recommended macro- and micronutrient intakes) and monitoring (growth and zinc, iron, vitamin B12, and vitamin D intake) should be undertaken by a health care professional” [61]. In line with the ESPGHAN, the Norwegian Directorate of Health advocates that a well-designed VN diet can be implemented for infants [62]. However, when addressing VN diets for infants, they refer to the positions of the ESPGHAN and AND, which also draw upon the guidance of the American Academy of Pediatrics on the use of VN formula for infants [62]. Spanish paediatricians, particularly the Committee on Nutrition and Breastfeeding of the Spanish Paediatric Association, released an elaborate narrative review as part of their position paper addressing the appropriateness of VG diets for infants and children. Their review considered viewpoints from the AND, ESPGHAN, and the Italian Society of Human Nutrition, along with original studies and numerous systematic reviews encompassing both child and adult research. Their position encompasses three key messages: (i) just like any diet, VG or VN diets should be carefully designed, (ii) following a VG diet at any age does not automatically imply danger, and (iii) it is advisable for infants and young children to follow a diet that includes a variety of foods, including animal products or, at the very least, an ovo-lacto-VG diet [63]. Finally, the Portugal National Programme for the Promotion of Healthy Eating also included an extensive literature review on the VG diet in their 2015 Guidelines for a Healthy Vegetarian Diet. However, they established their foundational position regarding VN diets for different demographic groups based on AND position statement papers from 2009, with most of the additional information derived from studies conducted on adult populations following VG and VN diets. They firmly continue from their foundation position, emphasizing that, when properly planned, VG diets—whether lacto-ovo-VG or VN—are healthy and nutritionally adequate during all stages of life. Furthermore, these diets can be helpful in both the prevention and treatment of some chronic diseases. Similar to any dietary pattern, VG diets may be inadequate [41].

Considering the growing popularity of VN diets for children, it would be rational to expect individual paediatric associations to include informative materials in their positions that aid parents in effectively implementing a carefully planned VN diet. This is particularly important, as only a limited number of nutritional guidelines address VN diets for the general population [64], often discussing them solely within the context of medically indicated diets or as alternative diets that are characterized by paediatric associations as unsuitable and unsafe for children [65,66,67]. To exemplify the perspective of the Slovenian Extended College of Paediatrics regarding VN diets for children, the position is closely linked to nutritional requirements within educational settings (i.e., public kindergartens and schools). They assert, without a literature review, that providing VN diets is not feasible for children and adolescents in these institutions [66,67]. However, it is essential to note that this standpoint does not extend to overseeing children’s dietary habits at home, including on afternoons, weekends, and holidays. The Slovenian Extended College of Paediatrics also states that the ESPGHAN guidelines for paediatric nutrition advise against vegan and caution vegetarian diets for children and adolescents [66], which needs to be corrected [3]. The 9-year-old Slovenian review paper about VG diets for children advocated that the medical profession does not recommend VN diets, and that they may not be advisable for children and adolescents [68]. They rely on the ESPGHAN publication from 2008, where the position was that infants and young children should not be fed VN diets [69]. Notably, their current position regarding VN diets for children lacks an increasing number of recent original references. Instead, they reference the ESPGHAN position in general without indicating the year of publication. Therefore, their current position [66,67] is still rooted in the outdated disapproval viewpoint from 15 years ago [69], without taking into account the more supportive recent references from 2011 [61] and 2017 [3]. In its latest position, the ESPGHAN specifically addresses VN diets within the framework of complementary feeding (i.e., from 4 to 6 months up to 12 months) [3]. The leading author of the ESPGHAN position statement, Fewtrell, M., is also a coauthor of a subsequent scientific publication, which discusses the consumption of plant-based diets in childhood to improve cardiometabolic health in adulthood [11]. This review and a 32-year follow-up of the CARDIA prospective study [70] suggest that plant-based diets adopted in childhood and young adulthood may have long-term benefits for cardiometabolic health. This includes the potential reduction of the risk of CVD from an early age and the promotion of overall longevity and well-being [11,70]. However, the authors stress the importance of further research to confirm the safety of providing predominantly or exclusively plant-based diets for children [11].

VN diets are a swiftly evolving field, coinciding with the growing number of children and parents adhering to them. If paediatric, dietetic, and nutritional societies/associations wish to keep their perspectives on VN diets, they will need to perform more frequent updates. Most position papers on the paediatric population need to be updated and incorporate recent findings concerning VN diets, which we present further in this review.

**Table 1:** Scientific/professional associations and expert groups that are supportive (S), reserved (R), or dismissive (D) regarding vegan nutrition during all life stages, including in pregnant women, breastfeeding women, infants, children, adolescents, adults, elderly individuals, and athletes (listed chronologically from 2003 to 2023).

Professional Associations and Expert Group	Year of Publication	S/R/D	Quoted Position
American Dietetic Association [58]	1997	S	Appropriately planned vegetarian diets are healthful, are nutritionally adequate, and provide health benefits in the prevention and treatment of certain diseases.
American Dietetic Association and Dietitians of Canada [46]	2003	S	Well-planned vegan and other types of vegetarian diets are appropriate for all stages of the life cycle, including during pregnancy, lactation, infancy, childhood, and adolescence.
European Society for Paediatric Gastroenterology, Hepatology, and Nutrition [69]	2008	D	Infants and young children receiving a vegetarian diet should receive a sufficient amount (500 mL) of milk (breast milk or formula) and dairy products. Infants and young children should not receive a vegan diet.
American Dietetic Association [45]	2009	S	Well-planned vegetarian diets are appropriate for individuals during all stages of the life cycle, including pregnancy, lactation, infancy, childhood, and adolescence, and for athletes.
Dietitians of Canada [42,71]	2010	S	A well-planned vegan diet can meet all of these needs. It is safe and healthy for pregnant and breastfeeding women, babies, children, teenagers, and seniors.
Ministry of Health of Slovenia [72]	2010	D	Vegan and macrobiotic foods are not suitable for children (of note: primarily related to infant diet).
Ministry of Health of Slovenia [73]	2010	D	It is not recommended for children and adolescents to eat a completely vegan diet, where meat and meat products, milk and milk products, and eggs are completely excluded from the diet. A vegan diet can be harmful to a child’s development and health and can lead to health consequences.
European Society for Paediatric Gastroenterology, Hepatology, and Nutrition [61]	2011	R	When a vegetarian diet is practiced, appropriate planning (taking into account recommended macro- and micronutrient intakes) and monitoring (growth, zinc, iron, vitamin B12, and vitamin D) should be undertaken by a health care professional (of note: their position refers for children aged 2–18 years).
National Institute of Public Health of Slovenia [74]	2011	D	We do not recommend a vegan diet, where meat and meat products, milk and milk products, and eggs are excluded from meals. A vegan diet can be harmful to a child’s development and health and can lead to serious health problems, which is why, based on numerous studies, we do not recommend it.
National Health and Medical Research Council of Australia [44]	2013	S	Appropriately planned vegetarian diets, including total vegetarian or vegan diets, are healthy and nutritionally adequate. Well-planned vegetarian diets are appropriate for individuals during all stages of the lifecycle.
Dietitians of Canada [75,76]	2014	S	A healthy vegan diet can meet all your nutrient needs at any stage of life including when you are pregnant, breastfeeding, or for older adults.
Extended Professional College for Paediatrics of Slovenia [67]	2015	D	The Extended Professional College for Paediatrics of Slovenia does not agree with the vegan diet for children and adolescents and does not support its introduction in kindergartens and schools.
Portugal National Programme for the Promotion of Healthy Eating [41]	2015	S	When appropriately planned, vegetarian diets, including lacto-ovo vegetarian or vegan, are healthy and nutritionally adequate for all cycles of life, and they can be useful in prevention and treatment of some chronic diseases.
Academy of Nutrition and Dietetics (before 2012 as American Dietetic Association) [43]	2016	S	It is the position of the Academy of Nutrition and Dietetics that appropriately planned vegetarian, including vegan, diets are healthful, nutritionally adequate, and may provide health benefits for the prevention and treatment of certain diseases.
German Nutrition Society [77]	2016	D	With a pure plant-based diet, it is difficult or impossible to attain an adequate supply of some nutrients. The most critical nutrient is vitamin B12. The DGE does not recommend a vegan diet for pregnant women, lactating women, infants, children, or adolescents.
Canadian Paediatric Society [78]	2017	S	Well-planned vegetarian diets (vegan diet included) can support pregnancy, breastfeeding and growth during infancy and childhood.
Italian Society of Human Nutrition [39]	2017	S	Well-planned vegetarian diets (lacto-ovo vegetarian and vegan) that include a wide variety of plant foods, and a reliable source of vitamin B12, provide adequate nutrient intake.
Italian Society of Preventive and Social Paediatrics, Italian Federation of Paediatricians, and Italian Society of Perinatal Medicine [79]	2017	D	A vegan diet should not be recommended for children because it lacks essential nutrients such as vitamin B12, DHA, iron, vitamin D, and calcium. Children following this diet should undergo careful monitoring of their growth and overall development.
European Society for Paediatric Gastroenterology, Hepatology, and Nutrition [3]	2017	S	Vegan diets should only be used under appropriate medical or dietetic supervision and parents should understand the serious consequences of failing to follow advice regarding supplementation of the diet (of note: their position refers to the framework of complementary feeding).
British Dietetic Association [38]	2017	S	Well-planned vegan diets can support healthy living in people of all ages.
Finish Food Authority [80]	2019	S	A carefully composed vegan diet is also suitable for pregnant and breastfeeding women, children, and young people. A vegan diet should be complemented with nutrition supplements that contain vitamin B12 and iodine.
Physicians Committee for Responsible Medicine [53,54]	2019	S	Vegan diets are appropriate, and they satisfy the nutrient needs and promote normal growth at all stages of the life cycle, including pregnancy and lactation, infancy, childhood, adolescence, older adulthood, and for athletes.
German Society for Paediatric and Adolescent Medicine [47]	2019	S	The nutritional needs of growing children and adolescents can generally be met through a balanced, vegetable-based diet. Vitamin B12 should be supplemented in people of all age groups who follow a strict vegan diet.
French Paediatric Hepatology, Gastroenterology and Nutrition Group [48]	2019	D	A vegan diet is not recommended for infants, children, and adolescents due to the risk of nutritional deficiencies that are inevitable in the absence of supplements. Vegan diets that exclude all animal products from the food register are not adapted to the human species.
Royal Academy of Medicine of Belgium [51]	2019	D	The commission considers that the vegan diet is unsuitable and therefore not recommended for unborn children, children, and adolescents, as well as pregnant and breastfeeding women.
Spanish Paediatric Association [63]	2020	R	A vegetarian or a vegan diet, as in any other kind of diet, needs to be carefully designed. After reviewing current evidence, even though following a vegetarian diet at any age does not necessarily mean it is unsafe, it is advisable for infant and young children to follow an omnivorous diet or, at least, an ovo-lacto-vegetarian diet.
Extended Professional College for Paediatrics of Slovenia [66]	2022	D	ESPGHAN guidelines for paediatric nutrition advise against vegan and caution vegetarian diets for children and adolescents.
British National Health Service [81]	2022	R	With good planning and an understanding of what makes up a healthy, balanced vegan diet, you can obtain all the nutrients your body needs. If you are bringing up your baby or child on a vegan diet, you need to ensure they obtain a wide variety of foods to provide the energy and vitamins they need for growth.
Dietitians Association of Australia [82]	2022	S	A varied and well-balanced vegetarian diet (vegan diet included) can supply all the nutrients needed for good health. Children need enough nutrients to help them grow and develop. Parents and carers of children following a vegetarian diet should take special care to ensure they are getting enough nutrition to thrive.
Norwegian Directorate of Health [62]	2022	S	With knowledge about diet and proper planning, it is possible to create a balanced and nutritious plant-based diet for infants (of note: their position refers to infant from 0 to 1 year of age).
Physicians Committee for Responsible Medicine [54,83]	2023	S	A plant-based diet is a healthful choice at every stage of life, including pregnancy and breastfeeding.
Kaiser Permanente [84]	2023	S	A well-planned vegetarian or vegan diet can be healthy for children and teens.

S: supportive, R: reserved, D: dismissive.

## 3. Studies on Vegan, Vegetarian, and/or Omnivorous Diets in Children and Adolescents

The Czech Vegan Children Study (CAROTS) included participants aged 0.5 to 18.5 years: 91 VG children (mean age 5.4 years), 75 VN children (mean age 4.4 years), and 52 OM children (mean age 6.7 years). It compared clinical, anthropometric, and blood/urine data on iodine status and thyroid function. Participants were recruited through general practitioners (OMs), social media, and VN-focused web pages (VNs). It was predicted that children on an OM diet recruited by general practitioners may have had a better-planned omnivore diet. In contrast, those on a VN diet recruited by VN-focused web pages might have had parents primarily motivated by factors other than health, potentially affecting the quality of their dietary choices. The results showed no significant differences in TSH, FT3, TG, or ATPOc levels among VG children, VN children, and OM children. Regarding iodine, OM children had the highest urinary iodine concentration (UIC), followed by VG and VN children. The median UIC in all groups exceeded the WHO’s 100 µg/L cut-off, although the recommended dietary intake of iodine was seldom met. Iodine deficiency was more prevalent in VN children (42%) and VG children (35%) than in OM children (20%) [85]. Height and body mass percentiles showed no significant differences among the groups, but more children in the VN group had low BMI. In another study with a similar sample size as the CAROTS (VG children = 79, VN children = 69, and OM children = 52, ranging from 0.5 to 18.5 years of age), no life-threatening vitamin B12 deficiency was detected among VNs with adequate supplementation, emphasizing the importance of supplementation in this group [86].

In the TARGet KIDS! longitudinal cohort study, 8907 children (average age at baseline of 2.2 years) were followed for approximately 2.8 years. Among these individuals, 248 were VG, including 25 VNs (10%) at the start of the study. Recruitment involved general practitioners, social media, VG-focused websites, and enrolment during routine health check-ups at 13 paediatric or family medicine clinics. The study aimed to explore the effects of a VG diet compared to an OM diet on growth, micronutrient stores (iron and vitamin D), and serum lipid levels in healthy children, considering factors such as cow’s milk consumption and age. The results indicated no significant associations between the consumption of a VG diet and zBMI, height-for-age z scores, and serum ferritin, 25-hydroxyvitamin D (25(OHD), and serum lipid levels. There were no clinically meaningful differences in growth or nutritional measures [87]. Two earlier comparative studies, conducted approximately 25–30 years ago on smaller sample sizes of VG children and OM children, yielded similar results regarding growth [88,89]. Notably, one of these studies, a UK longitudinal study of VG and OM children aged 7–11 years, found that VG children were more likely to be breastfed than OM children. However, the study acknowledged a limitation in not assessing the detailed quality of the VG diet, which could offer more insights into the observed outcomes [89]. In a recent cross-sectional study in Norway involving adults with lacto-lacto-VG, pesco-VG, and VN diets (*n* = 170), subclinical hypothyroidism (elevated TSH) was investigated. Notably, Norway lacks mandatory dietary iodine fortification. The results showed a low prevalence of subclinical hypothyroidism (3%), with no significant differences among dietary groups [90]. This rate was notably lower than that observed among adult Europeans (presumably following an OM diet) and Americans [91,92], aligning with findings from the Adventist Health Study-2, which also found no association of the consumption of a VN diet with hypothyroidism [93]. Given potential iodine intake concerns in VN diets, it is essential to provide practical guidelines for achieving recommended iodine levels [23,94]. Globally, iodine deficiency disorder remains a significant nutritional challenge affecting individuals of all demographics [95,96].

In Finland, a cross-sectional study evaluated 40 children (10 VG children, 6 VN children, 24 OM children) with an average age of 3.5 years. VN children had similar total fat, vitamin B12 (without supplementation), and calcium intake but lower protein, SFA, and cholesterol intake than OM children. On the other hand, VN children consumed more fibre (93%), folate (277%), iron (61%), and zinc (14%, without supplementation) than OM children. Both groups had similar intakes of vitamin D (with and without supplementation) and iodine (with iodized salt supplementation). However, both groups showed inadequate intake of eicosapentaenoic acid (EPA) and DHA without supplementation (OM children: 156 mg/day vs. VN children: 0 mg/day), and iron stores were comparable (16 µg/L vs. 14 µg/L). Additionally, there were no differences in height, BMI, or mid–upper arm circumference z scores between the dietary groups. Notably, the total number of children following a VN diet was four times smaller than that following an OM diet. Moreover, VN children had significantly lower total cholesterol (−32%), LDL cholesterol (−55%), and HDL cholesterol levels (1.4 mmol/L vs. 1.2 mmol/L) than OM children [97].

In a recent cross-sectional study of 187 Polish children aged 5–10 years (average 7.6 years), including VG children (63), VN children (52), and OM children (72), the risk of B12 deficiency was indeed higher in children from the VN group but not in those in the group supplemented with vitamin B12. The prevalence of possible B12 deficiency was 16% in OM children, 19% in VG children, and 40% in VN children. Mild and moderate iron deficiency anaemia was found in 2% of both VN and VG children and 6% and 7% of the sample, respectively, with no cases in OM children. Depleted iron stores were found in 13% of OM children, 18% of VG children, and 30% of VN children. Dietary intake differences were observed between the VN and OM groups. To assess dietary intake, the researcher employed food diaries over four consecutive days, encompassing two weekend days. VN children had a higher intake of carbohydrates (+23%), fibre (+200%), polyunsaturated fatty acids (PUFA) (+190%), beta carotene equivalents (+200%), folate (+180%), magnesium (+180%), and vitamin C (+74%) but a lower intake of protein (−36%), total fat (−27%), SFAs (−240%), cholesterol (−480%), monounsaturated fats (−28%), calcium (−45%), and vitamin B12 (−220%). Both groups had comparable energy intake and insufficient vitamin D intake (1.1 vs. 2.7 µg/d). However, on average, 15-hydroxyvitamin D levels were within the reference range (67 nmol/L vs. 57 nmol/L for OM children and VN children, respectively). Furthermore, the study indicated that a VN diet was associated with a healthier body composition and CVD risk profile. Abnormal and borderline high LDL-cholesterol levels were observed in 13% and 17% of OM children, 6% and 10% of VG children, and 0% and 1% of VN children, respectively. In addition, 40% more VN children had acceptable LDL-cholesterol levels (98.5% vs. 78.5%). However, differences in growth (with VN children being lighter and 3 cm shorter), with less body fat and comparable lean muscle mass and bone mineral content (VN children had 4–6% lower bone mineral content) were reported [96]. These differences could be attributed to the absence of cow’s milk and dairy products in VN diets, potentially linked to increased insulin-like growth factor 1 (IGF-1) [87,98,99,100]. However, taller stature has been associated with an increased risk of certain cancers [101,102,103,104], and greater height in adulthood resulting from higher milk intake in adolescence has been linked to a higher risk of bone fractures [105]. The study recruited VN and VG children through online platforms, suggesting that nutrition experts might not have optimally supervised their diets [98]. Enhanced guidance on well-designed VN diets for this specific age group, including recommendations for supplementation, could be beneficial.

In a recent cross-sectional study in Norway (*n* = 52) and Spain (*n* = 207) among children aged 6–13 years both with and without familial hypercholesterolemia (FH), researchers evaluated diet and lipid levels. Norwegian children used precoded food diaries validated by the University of Oslo, while Spanish children used a validated 137-item food frequency questionnaire from the PREDIMED study. Children from both countries primarily followed an OM diet. Regardless of FH status, Norwegian children had inadequate fibre intake, with a 25–35% lower intake, and Spanish children had a 24–29% lower intake compared to reference ranges that varied between the two countries. All groups exceeded the upper limit for SFA intake, with Norwegian children surpassing it by 71%. In addition, study participants had substantially lower-than-recommended MUFA (>50% lower in Norwegian children, 36% lower in Spanish children) and PUFA intake (almost 60% and 50% lower in Norwegian and Spanish children, respectively). The FH groups had higher total and LDL-cholesterol levels than the non-FH groups, while triglyceride levels were similar. The non-FH groups generally had lipid profiles within the recommended ranges. However, it is essential to note that the study did not include data on the children’s BMIs [106]. This study underscores the importance of well-designed diets, as even children consuming OM diets often fail to meet the gold standard regarding nutrient intake.

In the cross-sectional VeChi Diet Study in Germany, 430 children aged 1–3 years (127 VG children, 139 VN children, and 164 OM children) were examined regarding anthropometric measurements related to dietary intake using 3-day weighted dietary records. Participants were recruited through various channels related to VG and VN diets. No significant differences were found in dietary intake (regarding energy intake and macronutrients) or anthropometric measurements among the three groups. A VN diet in early childhood can support normal growth and nutritional needs comparably to an OM diet. Interestingly, children on a VN diet consumed significantly less added sugar (46% less than children on an OM diet in the basic model) and significantly more fibre (48% more than children on an OM diet). The authors concluded that both VG and VN diets in early childhood can support normal growth and nutritional needs comparable to an OM diet. Furthermore, the breastfeeding duration was longer in VN infants than in OM infants (15.9 vs. 11.1 months). On average, the parents of OM children were overweight (parents of OM children: BMI = 25.7 kg/m^2^ vs. parents of VN children: BMI = 24.5 kg/m^2^) [107]. In the same study, the authors assessed selenium intake, a critical nutrient for VG children and VN children, and compared selenium intake in the VG and VN groups with that in the OM group. On average, all three groups met the selenium requirements. However, as stated by the authors, 36% of VN children and 16% of OM children had inadequate selenium intake according to EFSA’s recommendation of 17 µg/day. Food sources rich in selenium for VN children include whole grains, nuts (especially Brazil nuts), and legumes. The study suggests that the risk of inadequate selenium intake among young VN children is generally low. Notably, none of the OM children reached the harmonized tolerable upper intake level of 60 µg/day, but 9% of VN children did. Adequate selenium intake is more related to dietary planning than dietary patterns [108].

The extensive VeChi Youth Study encompassing 401 children and adolescents aged 6 to 18 years (VG group = 149, VN group = 115, and OM group = 137) assessed dietary intake using 3-day weighted dietary records. In the VN group, higher intakes of carbohydrates (+15%), PUFA (+79%), fibre (+82%), magnesium (+64%), iron (+61%), vitamin E (+60%), vitamin C (+52%), folate (+39%), and vitamin B1 (+30%) compared to the OM group were recorded. In contrast, the OM group had higher intake levels of protein (+17%: 1.36 g/kg/day vs. 1.16 g/kg/day), free sugars (+59%), total fat (+24%), SFAs (+200%), monounsaturated fats (+24%), vitamin B_2_ (+43%), calcium (+31%), and, notably, a significantly higher vitamin B12 intake compared to the VN group. The energy, zinc, and vitamin intake of the groups were similar. Serum levels of haemoglobin, vitamin B_2_, 25(OH)D, folate, and methylmalonic acid showed no significant differences. In addition, 25(OH)D and vitamin B_2_ biomarker concentrations were low in a notable proportion of the study sample, independent of dietary group. Furthermore, the ferritin concentration was notably higher in the OM group (29 µg/L vs. 38 µg/L), but the status of both groups was within the reference range. Notably, the VN group exhibited significantly lower levels of total cholesterol (−20%), non-HDL cholesterol (−23%), LDL-cholesterol (−32%), and triglycerides (−15%). There were no differences detected in the attained body height. The study concluded that a VN diet can provide comparable dietary intake to an OM diet without specific nutritional risks and can support normal growth. Notably, more children on the OM diet were from families with a higher income status (81% vs. 62%) [109]. Moreover, financial limitations may not necessarily hinder the adoption of a plant-based dietary pattern [110].

In the Farm Study conducted between 1980 and 1983, researchers examined 404 children aged four months to 10 years, among which 14% were VG children, 93% were VN children, and 3% were OM children. During the first three years, the entire study population showed a slight decrease in bone height (0.2–2.1 cm) and body mass (0.1–1.1 kg), partly attributed to reference growth irregularities. However, after the age of five years, there were no significant differences in height, and by the age of ten years, VG children had an average height within 0.7 cm and body mass within 1.1 kg of the reference population. However, the authors did not differentiate further among the obtained results for VG and VN children. Most of these children followed a VN diet and achieved adequate growth, although their height and body mass were slightly lower than those of the reference population, with no marked abnormalities. Notably, two thirds of the group used vitamin/mineral supplements, and 95% were breastfed during the study [109]. It is important to note that, during the study, parents needed more comprehensive information and support for VN diets compared to recent times. Currently, VN individuals have access to various less healthy VN food options, often dense in energy, fat, free sugars, and salt, potentially affecting the nutritional profile of VN diets. A recent US cross-sectional study from 2001 to 2016 evaluated the diets of 9848 children aged 1–6 years by two 24 h dietary recall interviews from the NHANES study. The findings in OM children were concerning, with high rates of various deficiencies observed: 99% had fibre deficiency, 87% had vitamin D deficiency (79% for 1–2-year-olds, 87% for 2–3-year-olds, and 91% for 4–6-year-olds), 69% had vitamin E deficiency, 58% had potassium deficiency, and 17% had calcium deficiency (30% for 4–6-year-olds). The average DHA consumption was well below the EFSA recommendation (70–100 mg/day) for almost all children (97–99%), while nearly 100% exceeded the recommended sodium intake. The proportion of children with inadequate vitamin D, potassium, and calcium intake increased with age [111]. Notably, the published data do not include information on BMI. However, given the concerning dietary intake results, it is cautiously inferred that a significant proportion of the studied paediatric population was overweight or obese. Another study found that a significant proportion of US individuals aged two years or older (*n* = 16,338) did not meet the minimum recommended intakes for various plant food groups (ranging from 58% to 99%) [112]. Alarming statistics for US adolescents (12–19 years of age) report that nearly 40% are overweight or obese, 53% have abnormal lipid levels, 18% have prediabetes, and 15% have elevated blood pressure [12]. However, a recent study of 452 Chinese children aged 6–9 years on an OM diet showed that strong adherence to a healthy plant-based diet improved body composition as assessed by dual-energy X-ray absorptiometry [113].

During a 4-week randomized pilot trial performed in Cleveland (OH, USA), 30 pairs of obese children with hypercholesterolemia aged 9–18 years and their parents were divided into two groups. One group followed a whole-food, plant-based (VN) diet with no added fat, while the other group adhered to American Heart Association (AHA) diet guidelines (30% of energy from fat, less than 7% of energy from SFAs, less than 1500 mg of salt, and less than 300 mg of cholesterol). Both groups received 2 h weekly nutritional education classes. Both diets induced favourable effects, but the VN dietary group showed changes that were pronounced by more than 2-fold, including a decrease in BMI/fat, systolic blood pressure, total cholesterol, LDL-cholesterol, high-sensitivity C-reactive protein (hs-CRP), insulin, and waist circumference. Furthermore, nutrient intake improved as children transitioned from the initial OM diets to the interventional VN (whole food, plant-based) and OM (AHA) diets. Children on the VN diet showed significantly reduced intake of total fat (by 3-fold), SFAs (from 12% to 4% of total energy intake), cholesterol, and sodium (by 67%) and increased fibre intake (by 200%). However, vitamin D intake needed to be improved before the study. Omega-3 fatty acid, calcium (inadequate before the intervention), and vitamin B12 intake decreased significantly. The diet in the AHA dietary group resembled the initial OM diet, characterized by high total fat, SFA, and sodium intake and low levels of vitamin B12, vitamin D, iron, and calcium. Children on the AHA diet had lower intakes of protein, total fat (by 46%), SFAs (from 11% to 8% of total energy), cholesterol, and sodium (by 67%). Unfortunately, they also experienced undesirable reductions in the intake of omega-3 fatty acids, vitamin B12, vitamin D, calcium, and iron [114]. In a subsequent 1-year prospective randomized trial, 32 pairs of children and their parents were included in each dietary group (25 on the VN diet with added vitamin B12 and vitamin D, 27 on the AHA diet, and 28 on the Mediterranean (MED) diet). All participants were in the same age range as the initial study and had a history of overweight/obesity and elevated cholesterol levels. All three dietary groups had improved body mass, systolic and diastolic blood pressure, total cholesterol, and low-density lipoprotein levels. However, at the 52-week mark, the VN and MED groups showed more significant reductions in total and LDL cholesterol, blood glucose, and a novel cardiovascular biomarker of oxidative stress (myeloperoxidase). The results also indicated that the VN group consumed significantly lower amounts of protein, total fat, saturated fat, and trans fats, as well as lower amounts of cholesterol, sodium, vitamin D, and vitamin B12, but higher amounts of carbohydrates, fibre, and potassium compared to the AHA or MED groups [115].

The latest nationally representative dietary survey in Slovenia (Si. Menu 2017/2018) involved 468 adolescents aged 10–17 years (mean age 13.4 years) following an OM diet based on two 24 h dietary recalls that revealed inadequate intake of several essential nutrients. Specifically, approximately 100% of study participants reported lower than the recommended vitamin D intake, 91% reported inadequate fibre intake, 88% reported inadequate folate intake, and 44% reported inadequate iron intake. Moreover, 38% of participants had lower than the recommended intake of vitamin B12. Among girls, iron and vitamin B12 deficiencies were even more prevalent, found in 73% and 52%, respectively [116,117,118,119,120]. In addition, 55% of the adolescents adhered to the recommended BMI, while 44% of female and male adolescents were overweight or obese. The initial nationwide study on dietary intake was evaluated through a food frequency questionnaire (*n* = 2224) and 3-day weighted dietary records (*n* = 191) and studied Slovenian OM adolescents aged 14–17 years. The subjects were recruited at regional health centres while awaiting their medical examination. The study revealed that adolescents exceeded the recommended intake for free sugars, SFAs, and sodium. However, their dietary intakes of fibre density (only girls), PUFA, folate, fluoride, calcium, and vitamin D were below the recommended levels [121]. Notably, adequate iodine intake was associated with high salt consumption, surpassing the recommended limit by 150%. This is notable, considering that table salt iodization was implemented in Slovenia to address iodine deficiency. The researchers acknowledge that the results obtained align with those of other studies in children and adolescents (i.e., from Spain, Turkey, Germany, and Denmark) who presumably followed an OM diet. These studies also indicated inadequate energy and intake of several nutrients (fibre, vitamin D, folate, and fluoride) and the excessive intake of others (i.e., free sugars and total fat) [122]. Another study involving high school adolescents (*n* = 342) aged 14–16 years who followed an OM diet used a 24 h dietary recall for micronutrient intake and a questionnaire on the frequency of dietary habits. The study revealed that the studied adolescents exceeded the recommended minimum sodium intake by 2- to 3-fold. In addition, the majority of adolescents did not meet the recommended intake of fruits, vegetables, and fish (only a third did), milk/dairy products (only 40% did), and grains (only 50% did). Notably, adolescents exceeded the recommended intake levels for meat/meat products (320%) and sweet/savoury snacks (435%). Furthermore, 69% of adolescents used dietary supplements [123]. Notably, in line with the latter study, another more extensive study involving high school students aged 14–19 years from 15 high schools (*n* = 1463) estimated that as many as 69% consumed dietary supplements. The most commonly consumed nutritional supplements among nonathletes included vitamins, minerals, proteins/amino acids, and fats/fatty acids [124]. In line with the observed dietary intake results, the prevalence of excess body mass and obesity in Slovenian youths consuming unhealthy OM diets has increased significantly over the past three decades [125,126]. The proportion of obese youths tripled for both sexes from 1989 to the late 2000s, while the proportion of overweight youths doubled [125]. Data from 1989 to 2019 also showed an increase in subcutaneous fat across all age groups and sexes, while the decline in childhood obesity rates observed after 2010 was mainly due to a reduction in lean body mass [126]. It is crucial to note that the quality of nutritional patterns in VN diets, often overlooked in many studies, must be critically evaluated more broadly—alongside the presented concerning findings in children consuming OM diets and the relationship with BMI status and other health aspects.

A recent systematic review of 30 studies published between 2000 and 2022 evaluated the nutrient intake and status of children and adolescents (aged 2–18 years) who consumed VG, VN, and OM diets. The results showed that, in all diets, there was a potential risk of inadequate intake of vitamin D, calcium, probably also of iodine, EPA, and DHA (limited data for a firm conclusion). Children consuming an OM diet had a risk of inadequate folate and vitamin E intake, and their mean fibre, SFA, and PUFA intakes also deviated from the recommendations. Children consuming a VG diet did not meet the recommended intake of fibre, SFAs, and possibly PUFAs, but their mean intakes were more favourable than those of children consuming an OM diet. Children consuming a VN diet were at risk for inadequate vitamin B12, iron, and zinc intake. The authors concluded that there are risks of nutritional inadequacies in all dietary groups. Increasing the consumption of a variety of nutrient-rich plant foods, in combination with food fortification and possibly supplementation, is recommended to ensure that children and adolescents have sustainable and nutritionally adequate diets [127].

In conclusion, the frequent criticisms concerning the heterogeneity of studies in the literature, coupled with repeatedly limited participant numbers and varying degrees of favourable outcomes, may introduce a vital bias in evaluating the safety and suitability of VN diets for paediatric populations. Nevertheless, it is essential to note that criticism regarding the scientific quality of the supportive studies could also be applied to OM dietary patterns. Furthermore, it is essential that researchers focus on the same set of outcomes when comparing diets. Separate assessments of so-called nutritional adequacy in one study and CVD and other common risk factors in another study should be replaced by analyses of possible consequences of unhealthy OM diets and VN diets. By doing so, we can expect a more comprehensive evaluation of the benefits and risks, recognizing that well-designed planning can mitigate potential risks. In principle, such an approach avoids premature labelling that a particular dietary pattern is relatively more unhealthy or inappropriate. Table 2 summarizes the most critical available research findings in this area, many of which are not typically included in the discussion of positions held by relevant associations or expert groups.

## 4. Additional Considerations

**Figure 1:**
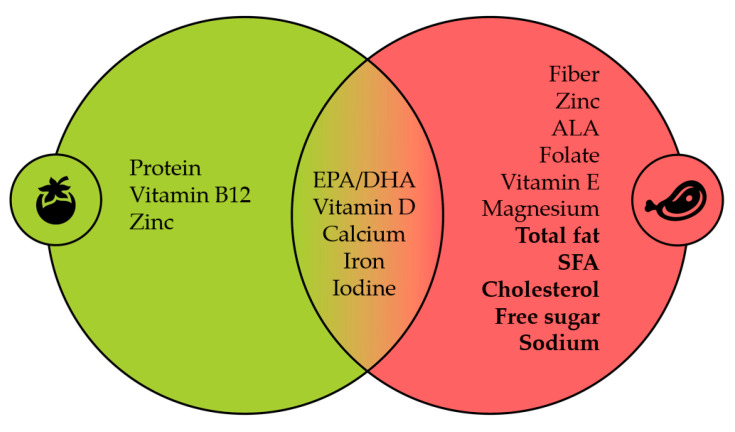
Green: VN diet, red: OM diet, nonbold: risk of inadequacy, bold: risk of excess intake. The data summarize the included studies and the latest systematic review [127].

In our review, we included the findings and short comments of studies cited by some relevant position papers on VN diets for children. These studies collectively play a vital role in providing a comprehensive context regarding the topic. They suggest that a poorly designed VN diet may lead to nutritional inadequacy, posing developmental risks for children. Such an argument extends beyond VN diets and could be applied to various dietary patterns, including OM diets. These studies emphasize an increased risk of nutritional inadequacies for specific nutrients such as EPA, DHA, vitamin B12, calcium, and zinc. However, they fail to mention that such a concern can similarly, if not more so, apply to OM diets and other nutrients like PUFA, ALA, fibre, folate, and magnesium. Furthermore, excessive nutrient intake (e.g., protein, total fat, SFAs, free sugars, and sodium) is also a shared concern. Figure 1 summarizes dietary intake outcomes primarily derived from paediatric studies.

Based on our present review of original studies, and in alignment with suggestions from various position papers addressing vitamin D intake, it is evident that the concern transcends dietary patterns alone. In this context, researchers focusing on position papers regarding VN diets for children should explicitly state that, while the dietary intake of vitamin D is frequently lower among VN children, typically, both groups fail to meet the reference levels when adhering to well-designed VN or OM diets.

Similarly, the studies included in our review emphasize the dietary intake of EPA and DHA in children on VN and OM diets as a central focus [97,111]. Indirect evidence from comparative studies exploring a variety of health effects of EPA and DHA in children and adults [129,130,131,132,133,134] suggests that lower EPA and DHA intake in VN children is not solely a matter of dietary choice. This notion is further supported by insights into the current low or inadequate consumption of fish and other marine sources among OM children [135,136,137,138] and broader considerations of environmental sustainability concerning fish and seafood intake to meet EPA and DHA recommendations [139]. These findings highlight that this issue extends beyond dietary choices. Based on these insights, we recommend that the authors of future position statement papers adopt a more scientifically balanced approach when addressing EPA and DHA nutrients within VN diets. In addition, future position statements regarding the suitability of VN diets for children should also include information on the increased occurrence of food allergies related to specific plant-based foods, the risks associated with the consumption of ultra-processed VN products, and guidance on dietary modifications to ensure continued nutritional adequacy [140,141,142]. For VN diets, it is often stated that the cost is high, and only individuals with higher economic status can afford to follow them. In contrast, studies on children, adolescents, and adults have shown that a VN diet can save on food costs [110,143,144].

Finally, regarding the aim of the narrative review (1–3), position statements and expert opinions regarding VN diets for children should be updated more regularly and properly cited; conclusions should consider comparative studies with children with OM diets and current public health challenges related to unhealthy diets and lifestyles.

## 5. Strengths and Limitation

Our narrative review offers a robust and comprehensive analysis of research on the nutrient intake status of children following VN and OM diets. We carefully examined the chronological listing of the position papers of relevant associations and their quoted positions and briefly analysed their references. Furthermore, we conducted an in-depth analysis of published nutritional studies on VN and OM diets for children; most of these studies were not included in previous position papers. To further enhance our review, we included a recently published systematic review of 30 studies examining the nutrient intake status of children who consumed VN, VG, and OM diets [127]. This nicely summarized a part (comparison between dietary patterns in terms of the risk of inadequacy and (un)favourably high intakes) of the issue that researchers in the literature often avoid, which distorts the conclusions. In addition, the reviewed studies primarily consisted of observational studies (except two), which limits the scope of our narrative review. Therefore, a recommendation in support of or against a VN diet compared with OM diet should be assessed carefully, without bias towards one diet or the other as a result of an eating preference, a past “indoctrinated business model”, or ignoring the total body of evidence; additionally, a recommendation should be made in the context of the existence of known public health challenges as a result of unhealthy diets and lifestyles. To achieve the best possible nutrition for children, we recommend following a VN or OM diet consisting of energetically and nutritionally sufficient whole foods to resolve nutritional disagreements until better scientific evidence for or against VN or OM diets for children is available.

## 6. Future Recommendations in the Area

Conducting well-designed comparative (long-term) studies is advisable to avoid unnecessary speculations regarding the suitability of VN diets for children. These studies should initially consider well-designed VN and OM diets and subsequently explore how these diets are implemented in practical settings. They should also monitor the effects of the different types of diets on nutrient intake and status by using biomarkers and investigating several functional outcomes, such as growth, development, and the prevention of chronic NCDs [127]. Swedish researchers have announced a long-term observational study, the Vegan Diet in Small Children (VHS) study, planned to be conducted from 2021 to 2026. This study will involve 30 children under six months of age whose parents have chosen exclusively to provide them with a VN diet. These children will be compared with a reference group of 30 matched cases following an OM diet. Data collection will include monthly dietary records, blood sample analyses, and growth measurements at 6, 12, and 24 months of age. At the age of 3 years, cognitive developmental assessments and DEXA body composition examinations will be conducted. The study aims to increase the understanding of the benefits and potential risks associated with both VN and OM diets, focusing on aspects such as growth, cognitive development, and nutritional status [145].

Regardless of minor variations in current position statements among the most relevant scientific/professional associations, there is a strong consensus that children’s diets, in general, should predominantly align with plant-based diets. Children should consume more whole grains, legumes, vegetables and fruits, and nuts and seeds to achieve adequate intakes of fibre, SFAs, PUFAs, folate, and vitamin E [127]. Such a consensus arises from the current health status of children and adults, understanding that the adoption of a plant-based eating pattern serves as an investment in not only the improvement in adult body mass and body composition management, but also in addressing the significant public health challenges regarding cardiometabolic health [11,12,70,125,126,146,147].

Finally, in a recent Italian survey, a notable challenge emerged, as paediatricians were perceived as sceptical and unprepared when they were asked about raising children on VN diets [21]. In situations where opportunities for improvement remain, such as ensuring that VN diets are well designed (i.e., that cover energy needs as well as macro and nutrient needs, without a high intake of sugar, salt, and SFAs) and practised appropriately (i.e., predominantly from whole foods, with adequate supplementation with vitamins B12 and D in winter months, probably also with EPA and DHA, and low intake of highly processed foods that are rich in sugar, salt, and preservatives) [23], it is paramount to recognize the significance of fostering effective communication among parents, paediatricians, and dietitians. Such a practice should stand as a fundamental cornerstone when devising nutritional strategies for the well-being of children adhering to VN diets [29].

## 7. Conclusions

Recent studies on children following VN (including VG) and OM diets coincide with the obesity epidemic among children and adults consuming unhealthy OM diets, the increasing prevalence of chronic NCDs, and the environmental challenges. The sceptical views surrounding VN diets for children in position papers are because the claims often do not rely on well-designed VN diets supplemented with vitamin B12 but mainly on findings in children who have not properly implemented a VN diet.

The health benefits in adults following VN diets, the fact that the position papers of numerous (paediatric) associations are poorly referenced, based on outdated or unrepresentative studies, often with limited information about VN diets, the supporting system, and the nutritional choices, all highlight the need for re-evaluation. Based on the current studies, a well-designed VN diet is not inherently appropriate and safe for children; therefore, an OM diet is not inherently superior, optimal, nor a gold standard. The authors of position statements should apply the same evaluation approach towards VN as well as OM diets.

We suggest that paediatric associations frequently update position papers and include the recent high-quality studies. Efforts should be made to improve both types of diets and to ensure they are well designed, consist primarily of whole foods, and are properly implemented. It is essential that children on a VN diet consume a variety of whole plant foods and/or foods that are appropriately fortified with critical micronutrients (i.e., vitamins B12 and D, calcium, iron, zinc, and iodine). If such foods are not consumed in adequate amounts, supplementation should be recommended. Position papers should also include information for paediatricians and dietitians on effective counselling for parents and offer practical information (i.e., brochures, cooking classes, recipes, lists of local farmers, web pages) on the preparation of healthy and sustainable meals for children. Position papers, health professionals, and media reports also play a vital role in ensuring that whole, nutritious plant-based foods and meals, including fruits, vegetables, legumes, whole grains, nuts, and seeds, become more affordable and broadly available, and are therefore regularly consumed as the new normal. A paramount focus should be placed on facilitating implementation, fostering an inclusive environment for parents and children, regardless of their dietary choices. This is particularly important because we live in an obesogenic food environment with increasing health care costs, where both diets can quickly be practised in an unhealthy manner.

## Figures and Tables

**Table 2:** Summaries of the specifically addressed relevant studies.

Country of the Study	Year of Publication/Years of Recruitment	Study Design	Number of Children	Age (Years)	Observed Parameters	Important Results	Important Features
Czech Republic [85]	2023/2019–2021	Cross-sectional	VG children = 91, VN children = 75, OM children = 52	0.5–18.5	Iodine status and thyroid function	No difference in thyroid function; the UIC was highest in the OMs; all groups exceeded the WHO cut-off; RDI was seldom met in all groups; higher number of VNs with lower BMIs.	VNs recruited through VN-focused web pages and OMs recruited by general practitioners.
Czech Republic [86]	2022/2019–2021	Cross-sectional	VG children = 79, VN children = 69, OM children = 52	0.5–18.5	Vitamin B12 intake and serum status	Life-threatening or severe laboratory-confirmed vitamin B12 deficiency was not detected among VNs; VNs were well supplemented.	VNs recruited through VN-focused web pages, OMs recruited by general practitioners.
Canada [87]	2022/2008–2019	Longitudinal cohort	VG children = 248, (VN children = 25), OM children = 8659	0.5–10	Anthropometric measurements (growth status), serum lipid, iron, and vitamin D levels	No difference in growth or biochemical measures; more VGs were underweight (2.9% absolute diff.) and more OMs were overweight (3.1% absolute diff.) and obese (0.9% absolute diff.); more families of OM children had higher incomes (43% vs. 27%).	VG group has 10% VNs and the impact on the results was not clear; no dietary intake assessment; clinical relevance of the small absolute diff. in body mass was not clear.
Finland [97]	2021/2017	Cross-sectional	VG children = 10, VN children = 6, OM children = 24	1–7	Metabolic and nutritional status	Diet: VNs had higher intakes of fibre, LA, ALA, MUFA, PUFA, vitamin B12 (with suppl.), folate, vitamin K, magnesium, and iron (without suppl.); VNs had lower intakes of protein, SFAs, cholesterol, and EPA/DHA and similar vitamin D (with and without suppl.), calcium, and iodine intake (with suppl.); blood: VNs had higher folate and ALA levels and lower total vitamin D, lipoprotein, and DHA levels.	The comparison included 4 times fewer VNs (only 6) than OMs; OMs consumed unusually low amounts of fish and fish dishes to obtain 156 mg of EPA/DHA without suppl. (2 g/day), vitamin B12 intake (without suppl.) was similar between VNs and OMs (3.2 µg/day vs. 3.5 µg/day).
Poland [98]	2021/2014–2016	Cross-sectional	VG children = 63, VN children = 52, OM children = 72	5–10	Body composition, CV, and nutritional risk	More VN children had vitamin B12 deficiency but not in the supplemented group; all groups had some prevalence of B12 deficiency anaemia and depleted iron stores, with more VNs; VNs had 2 times higher fibre and 1.8 times higher magnesium intake but lower protein, total fat, SFA, cholesterol, MUFA, and calcium intake; both groups had inadequate vitamin D intake but within the reference mean D(25)OH level; OMs had a higher prevalence of abnormal and borderline high LDL-cholesterol, and higher hs-CRP levels; VNs were lighter (BMI), shorter (by 3 cm), and had lower fat mass index (but not lean mass index), with 4–6% lower bone mineral content.	VNs recruited through VN-focused web pages, OMs recruited by asking VNs and VGs to invite a friend.
Norway/Spain [106]	2021/2013–2015	Cross-sectional	OM children _(Norway)_ = 52, OM children _(Spain)_ = 207	6–13	Dietary intake and lipid levels	Inadequate intake of fibre, MUFA, and PUFA and exceeded intake of SFAs; lipid profiles of both non-FH cohorts were within the recommended levels.	No BMI status reported.
Germany [109]	2021/2017–2021	Cross-sectional	VG children = 149, VN children = 115, OM children = 137	6–18	Dietary intake, anthropometric measurements, and nutritional status	VN diet can provide a comparable dietary intake and support normal growth; VNs: higher intake of carbohydrates, PUFA, fibre, magnesium, iron, vitamin C, folate, and vitamin B1; OMs: higher intake of protein, free sugar, total fat, SFA, MUFA, vitamin B2, calcium; no diff. in serum: Hb, vitamin B2, 25 (OHD, folate, and methylmalonic acid.	Ferritin levels were higher in OMs, but both group’s statuses were within the reference range; 25(OH)D and vitamin B2 intake was low in a notable proportion of individuals, independent of diet; more OM children belonged to families with higher incomes (81% vs. 62%).
Germany [107]	2019/2016–2018	Cross-sectional	VG children = 127, VN children = 139, OM children = 164	1–3	Dietary intake and anthropometric measurements	No diff. in energy and macronutrient intake nor anthropometric measurements; higher % of OMs were overweight or at possible risk of overweight; 3.6% of VNs were classified as overweight; VN infants were breastfed 43% longer.	VNs consumed (basic model) 46% less added sugar, 13% less protein (but 2.4 times higher than are the reference), and 48% more fibre than OMs; parents’ primary motive for VN diets was ethical reasons (68%).
Germany [108]	2019/2016–2018	Cross-sectional	VG children = 127, VN children = 139, OM children = 164	1–3	Selenium intake differences	All groups met the selenium requirements (17 µg/day); more VNs fell below the EFSA reference (36% vs. 16%).	Clinical relevance of lower selenium intake in VNs was uncertain and the risk of inadequate intake was generally low; harmonized tolerable upper intake level (60 µg/day) was not reached by OMs but was reached by 9% of VNs.
USA [111]	2021/2001–2016	Cross-sectional	OM children = 9848	1–6	Nutrient intake	Proportion of children with inadequate intake: fibre (99%), DHA (97–99%), vitamin D (87%), vitamin E (69%), potassium (58%), and calcium (17%); exceeded intake of sodium (nearly 100%).	The proportion of children with inadequate intake of vitamin D, potassium, and calcium increased with age; no BMI status reported.
USA [115]	2021/2016–2017	Prospective randomized 1 year intervention	VN = 25, AHA = 27, MED = 28	9–18	Cardiovascular risk and dietary intake change	The VN and MED groups showed greater reductions in body mass, total cholesterol, LDL cholesterol, glucose, and amyeloperoxidase; the VN group consumed lower amounts of protein, total cholesterol, SFAs and trans fats, cholesterol, sodium, vitamin D, and vitamin B12, but higher amounts of carbohydrates, fibre, and potassium compared to the AHA or MED groups.	The nutritional adequacy of all three diets was not reported.
USA [114]	2015/2013	Prospective randomized 4-week intervention	VG children = 14, AHA children = 14	9–18	Cardiovascular risk and dietary intake change	Both dietary groups (VN = WFPB and OM = AHA) showed favourable effects, but the VN dietary group fared better in terms of decreased BMI/fat, systolic blood pressure, total and LDL-cholesterol, hs-CRP, insulin, and waist circumference; both groups showed improvements (less SFA and sodium, more fibre), but still had inadequate intake of vitamin B12, vitamin D, calcium, and iron.	The intervention period was short; children were mostly in obese (71% on a VN diet and 86% on an OM diet).
USA [128]	1989/1980–1983	Cross-sectional	VG children = 64, VN children = 288, OM children = 28	4 months to 10 years	Growth	VG children had an average height within 0.7 cm and body mass of 1.1 kg of the reference; authors: children on VN diets can achieve adequate growth.	No further differentiation in the obtained results between VGs and VNs; a small proportion of children on the OM diet (3%); the obtained data are 40 years old.
Slovenia [116,117,118,119]	2021–2022/2017	Cross-sectional	OM children = 468	10–17	Dietary intake and BMI status	Inadequate intake of fibre (19.5 g/day, 91% when the cut-off value was 30 g/day), vitamin B12 (5.4 µg/day, 47% when the cut-off was <4 µg/day), vitamin D (2.7 µg/day, 100% when the cut-off was 20 µg/day), folate (290 µg/day, 88% when the cut-off was <400 µg/day), iron (17 mg/day, 44% when the cut-off was 12 µg/day); 44% were overweight or obese.	Females: inadequate intake of iron (73%) and vitamin B12 (52%).
Slovenia [123]	2019/2014	Cross-sectional	OM children = 343	14–16	Eating habits and micronutrient intake	Inadequate intake: only 30% for fruits, vegetables, and fish, 40% for milk/dairy products, and 50% for cereals/cereals products; exceeded intake: 320% for meat/meat products, 453% for sweet/savoury snacks); micronutrient intake: exceeded sodium intake by 2–3 times.	69% of adolescent used dietary supplements; for micronutrient intake, the authors used a less precise one-time 24 h recall; no BMI status reported.
Slovenia [121,122]	2009, 2012/2003–2005	Cross-sectional	OM children = 2224/2485	15–16	Dietary (macro- and micronutrients) intake	Below the recommendations: fibre density (only girls), PUFA, folate, fluoride, calcium, and vitamin D (4 µg/day); exceeded intake of free sugars (16% of E), SFAs (13% of E), and sodium (>4000 mg/day).	The dietary habits of Slovenian adolescents were less than optimal. They exceeded the reference intake values for free sugars, salt, and SFAs and consumed below the reference intake for PUFA, water, and several micronutrients. Adequate iodine intake was associated with excess salt intake (150% above the recommended limit).

## Data Availability

Not applicable.

## References

[1] De Cosmi V., Scaglioni S., Agostoni C. (2017). Early taste experiences and later food choices. Nutrients.

[2] Waterland R.A., Garza C. (1999). Potential mechanisms of metabolic imprinting that lead to chronic disease. Am. J. Clin. Nutr..

[3] Fewtrell M., Bronsky J., Campoy C., Domellöf M., Embleton N., Fidler Mis N., Hojsak I., Hulst J.M., Indrio F., Lapillonne A. (2017). Complementary Feeding: A Position Paper by the European Society for Paediatric Gastroenterology, Hepatology, and Nutrition (ESPGHAN) Committee on Nutrition. J. Pediatr. Gastroenterol. Nutr..

[4] Tran B.X., Dang K.A., Le H.T., Ha G.H., Nguyen L.H., Nguyen T.H., Tran T.H., Latkin C.A., Ho C.S.H., Ho R.C.M. (2019). Global evolution of obesity research in children and youths: Setting priorities for interventions and policies. Obes. Facts.

[5] Di Cesare M., Sorić M., Bovet P., Miranda J.J., Bhutta Z., Stevens G.A., Laxmaiah A., Kengne A.P., Bentham J. (2019). The epidemiological burden of obesity in childhood: A worldwide epidemic requiring urgent action. BMC Med..

[6] Jing L., Binkley C.M., Suever J.D., Umasankar N., Haggerty C.M., Rich J., Nevius C.D., Wehner G.J., Hamlet S.M., Powell D.K. (2016). Cardiac remodeling and dysfunction in childhood obesity: A cardiovascular magnetic resonance study. J. Cardiovasc. Magn. Reson..

[7] Napoli C., D’Armiento F.P., Mancini F.P., Postiglione A., Witztum J.L., Palumbo G., Palinski W. (1997). Fatty streak formation occurs in human fetal aortas and is greatly enhanced by maternal hypercholesterolemia. Intimal accumulation of low density lipoprotein and its oxidation precede monocyte recruitment into early atherosclerotic lesions. J. Clin. Investig..

[8] Skilton M.R., Siitonen N., Würtz P., Viikari J.S.A., Juonala M., Seppälä I., Laitinen T., Lehtimäki T., Taittonen L., Kähönen M. (2014). High birth weight is associated with obesity and increased carotid wall thickness in young adults: The cardiovascular risk in young Finns study. Arterioscler. Thromb. Vasc. Biol..

[9] McGill H.C., Herderick E.E., McMahan C.A., Zieske A.W., Malcom G.T., Tracy R.E., Strong J.P. (2002). Atherosclerosis in youth. Minerva Pediatr..

[10] Strong J.P., Malcom G.T., McMahan C.A., Tracy R.E., Newman W.P., Herderick E.E., Cornhill J.F. (1999). Prevalence and extent of atherosclerosis in adolescents and young adults: Implications for prevention from the Pathobiological Determinants of Atherosclerosis in Youth Study. JAMA.

[11] Desmond M.A., Sobiecki J., Fewtrell M., Wells J.C.K. (2018). Plant-based diets for children as a means of improving adult cardiometabolic health. Nutr. Rev..

[12] Perak A.M., Baker-Smith C., Hayman L.L., Khoury M., Peterson A.L., Ware A.L., Zachariah J.P., Raghuveer G., American Heart Association Council on Hypertension, Council on Cardiovascular and Stroke Nursing (2023). Toward a Roadmap for Best Practices in Pediatric Preventive Cardiology: A Science Advisory From the American Heart Association. Circ. Cardiovasc. Qual. Outcomes.

[13] Termannsen A.D., Clemmensen K.K.B., Thomsen J.M., Nørgaard O., Díaz L.J., Torekov S.S., Quist J.S., Færch K. (2022). Effects of vegan diets on cardiometabolic health: A systematic review and meta-analysis of randomized controlled trials. Obes. Rev..

[14] Koch C.A., Kjeldsen E.W., Frikke-Schmidt R. (2023). Vegetarian or vegan diets and blood lipids: A meta-analysis of randomized trials. Eur. Heart J..

[15] Pollakova D., Andreadi A., Pacifici F., Della-Morte D., Lauro D., Tubili C. (2021). The Impact of Vegan Diet in the Prevention and Treatment of Type 2 Diabetes: A Systematic Review. Nutrients.

[16] Dinu M., Abbate R., Gensini G.F., Casini A., Sofi F. (2017). Vegetarian, vegan diets and multiple health outcomes: A systematic review with meta-analysis of observational studies. Crit. Rev. Food Sci. Nutr..

[17] Scarborough P., Clark M., Cobiac L., Papier K., Knuppel A., Lynch J., Harrington R., Key T., Springmann M. (2023). Vegans, vegetarians, fish-eaters and meat-eaters in the UK show discrepant environmental impacts. Nat. Food.

[18] Chai B.C., van der Voort J.R., Grofelnik K., Eliasdottir H.G., Klöss I., Perez-Cueto F.J.A. (2019). Which Diet Has the Least Environmental Impact on Our Planet? A Systematic Review of Vegan, Vegetarian and Omnivorous Diets. Sustainability.

[19] Ripple W.J., Wolf C., Newsome T.M., Barnard P., Moomaw W.R. (2019). World Scientists’ Warning of a Climate Emergency. Bioscience.

[20] Mahase E. (2021). What does the evidence say about vegan diets in children?. BMJ.

[21] Bivi D., Di Chio T., Geri F., Morganti R., Goggi S., Baroni L., Mumolo M.G., de Bortoli N., Peroni D.G., Marchi S. (2021). Raising Children on a Vegan Diet: Parents’ Opinion on Problems in Everyday Life. Nutrients.

[22] Benedik E., Fidler Mis N. (2013). New recommendations for vitamin D intake. Slov. J. Public Health.

[23] Jakše B. (2021). Placing a Well-Designed Vegan Diet for Slovenes. Nutrients.

[24] Baldassarre M.E., Panza R., Farella I., Posa D., Capozza M., Di Mauro A., Laforgia N. (2020). Vegetarian and Vegan Weaning of the Infant: How Common and How Evidence-Based? A Population-Based Survey and Narrative Review. Int. J. Environ. Res. Public Health.

[25] Hall K.D. (2018). Did the Food Environment Cause the Obesity Epidemic?. Obesity.

[26] Gearhardt A.N., Hebebrand J. (2021). The concept of “food addiction” helps inform the understanding of overeating and obesity: Debate Consensus. Am. J. Clin. Nutr..

[27] Fidler Mis N., Braegger C., Bronsky J., Campoy C., Domellöf M., Embleton N.D., Hojsak I., Hulst J., Indrio F., Lapillonne A. (2017). Sugar in Infants, Children and Adolescents: A Position Paper of the European Society for Paediatric Gastroenterology, Hepatology and Nutrition Committee on Nutrition. J. Pediatr. Gastroenterol. Nutr..

[28] Sutter D.O., Bender N. (2021). Nutrient status and growth in vegan children. Nutr. Res..

[29] Kostecka M., Kostecka J., Jackowska I., Iłowiecka K. (2023). Parental Nutritional Knowledge and Type of Diet as the Key Factors Influencing the Safety of Vegetarian Diets for Children Aged 12–36 Months. Nutrients.

[30] Menzel J., Abraham K., Stangl G.I., Ueland P.M., Obeid R., Schulze M.B., Herter-Aeberli I., Schwerdtle T., Weikert C. (2021). Vegan Diet and Bone Health—Results from the Cross-Sectional RBVD Study. Nutrients.

[31] Tong T.Y.N., Appleby P.N., Armstrong M.E.G., Fensom G.K., Knuppel A., Papier K., Perez-Cornago A., Travis R.C., Key T.J. (2020). Vegetarian and vegan diets and risks of total and site-specific fractures: Results from the prospective EPIC-Oxford study. BMC Med..

[32] Gilsing A.M.J., Crowe F.L., Lloyd-Wright Z., Sanders T.A.B., Appleby P.N., Allen N.E., Key T.J. (2010). Serum concentrations of vitamin B12 and folate in British male omnivores, vegetarians and vegans: Results from a cross-sectional analysis of the EPIC-Oxford cohort study. Eur. J. Clin. Nutr..

[33] Spencer E.A., Appleby P.N., Davey G.K., Key T.J. (2003). Diet and body mass index in 38 000 EPIC-Oxford meat-eaters, fish-eaters, vegetarians and vegans. Int. J. Obes..

[34] Webster J., Greenwood D.C., Cade J.E. (2023). Risk of hip fracture in meat-eaters, pescatarians, and vegetarians: A prospective cohort study of 413,914 UK Biobank participants. BMC Med..

[35] Barnard N.D., Willet W.C., Ding E.L. (2017). The Misuse of Meta-analysis in Nutrition Research. JAMA.

[36] Li T., Li Y., Wu S. (2021). Comparison of human bone mineral densities in subjects on plant-based and omnivorous diets: A systematic review and meta-analysis. Arch. Osteoporos..

[37] Bala M., Kashuk J., Moore E.E., Catena F., Leppaniemi A., Ansaloni L., Biffl W., Coccolini F., Peitzman A., Sartelli M. (2018). Establishing position papers by the WSES. World J. Emerg. Surg..

[38] The Association of UK Dietitians British Dietetic Association Confirms Well-Planned Vegan Diets Can Support Healthy Living in People of All Ages. https://www.bda.uk.com/news/view?id=179.

[39] Agnoli C., Baroni L., Bertini I., Ciappellano S., Fabbri A., Papa M., Pellegrini N., Sbarbati R., Scarino M.L., Siani V. (2017). Position paper on vegetarian diets from the working group of the Italian Society of Human Nutrition. Nutr. Metab. Cardiovasc. Dis..

[40] Craig W.J., Mangels A.R., Fresán U., Marsh K., Miles F.L., Saunders A.V., Haddad E.H., Heskey C.E., Johnston P., Larson-meyer E. (2021). The Safe and Effective Use of Plant-Based Diets with Guidelines for Health Professionals. Nutrients.

[41] Gomes S.C., João S., Pinho P., Borges C., Santos C.T., Santos A., Graça P. (2015). National Programme for the Promotion of Healthy Eating Guidelines for a Healthy Vegetarian Diet.

[42] Amit M., Cummings C., Grueger B., Feldman M., Lang M., Grabowski J., Wong D., Greig A., Patel H. (2010). Vegetarian diets in children and adolescents. Paediatr. Child Health.

[43] Melina V., Craig W., Levin S. (2016). Position of the Academy of Nutrition and Dietetics: Vegetarian Diets. J. Acad. Nutr. Diet..

[44] National Health and Medical Council of Australia Australian Dietary Guidelines. https://www.nhmrc.gov.au/about-us/publications/australian-dietary-guidelines.

[45] Craig W.J., Mangels A.R. (2009). Position of the American Dietetic Association: Vegetarian diets. J. Am. Diet. Assoc..

[46] American Dietetic Association, Dietitians of Canada (2003). Position of the American Dietetic Association and Dietitians of Canada: Vegetarian diets. J. Am. Diet. Assoc..

[47] Rudloff S., Bührer C., Jochum F., Kauth T., Kersting M., Körner A., Koletzko B., Mihatsch W., Prell C., Reinehr T. (2019). Vegetarian diets in childhood and adolescence. Mol. Cell. Pediatr..

[48] Lemale J., Mas E., Jung C., Bellaiche M., Tounian P. (2019). Vegan diet in children and adolescents. Recommendations from the French-speaking Pediatric Hepatology, Gastroenterology and Nutrition Group (GFHGNP). Arch. Pediatr..

[49] Roed C., Skovby F., Meldgaard A. (2009). Severe vitamin B12 deficiency in infants breastfed by vegans. Ugeskr. Laeger.

[50] Larsson C.L., Johansson G.K. (2002). Dietary intake and nutritional status of young vegans and omnivores in Sweden. Am. J. Clin. Nutr..

[51] Académie Royale de Médecine de Belgique Régimes Végétariens et Végétaliens Administrés aux Enfants et Adolescents. https://gastronomiaycia.republica.com/wp-content/uploads/2019/05/Informe_academia_belgica_1.pdf.

[52] Martinez-Biarge M. A Letter of Protest of Plant-Based Health Professionals UK. https://creciendoenverde.com/wp-content/uploads/2019/07/Vegan-diets-in-Children_Full-response-to-Prof-Casimir_20.06.19.pdf.

[53] Barnard N., Levin S., Kahleova H., Melina V., Mangels R., Craig W. Letter of Protest of Physicians Committee for Responsible Medicine. https://www.pcrm.org/sites/default/files/2019-05/Letter-to-Belgian-Academie-2019-05-22-RM-VM-WC.pdf.

[54] Barnard N., Levin S., Kahleova H., Vesanto M., Mangels R., Craig W.U.S. Doctors Blast Belgian Misinformation on Vegan Diets. https://www.pcrm.org/news/news-releases/us-doctors-blast-belgian-misinformation-vegan-diets.

[55] Lin Y., Huybrechts I., Vandevijvere S., Bolca S., De Keyzer W., De Vriese S., Polet A., De Neve M., Van Oyen H., Van Camp J. (2011). Fibre intake among the Belgian population by sex–age and sex–education groups and its association with BMI and waist circumference. Br. J. Nutr..

[56] Deriemaeker P., Alewaeters K., Hebbelinck M., Clarys P. (2011). Nutritional intake of various groups of Flemish vegetarians. Arch. Public Health.

[57] Chini M. Flemish Millennials Told to Eat More Vegetables. https://www.brusselstimes.com/55552/flemish-millennials-told-to-eat-more-vegetables.

[58] Messina V.K., Burke K.I. (1997). Position of the American dietetic association: Vegetarian diets. J. Am. Diet. Assoc..

[59] Kleinman R.E., American Academy of Pediatrics, Committee on Nutrition (2008). Pediatric Nutrition Handbook.

[60] Merritt R.J., Fleet S.E., Fifi A., Jump C., Schwartz S., Sentongo T., Duro D., Rudolph J., Turner J. (2020). North American Society for Pediatric Gastroenterology, Hepatology, and Nutrition Position Paper: Plant-based Milks. J. Pediatr. Gastroenterol. Nutr..

[61] Agostoni C., Braegger C., Decsi T., Kolacek S., Koletzko B., Mihatsch W., Moreno L.A., Puntis J., Shamir R., Szajewska H. (2011). Role of dietary factors and food habits in the development of childhood obesity: A commentary by the ESPGHAN Committee on Nutrition. J. Pediatr. Gastroenterol. Nutr..

[62] Norwegian Directorate of Health Vegetarian and Vegan Food for Infants (0–1 Years of Age). https://www.helsenorge.no/en/kosthold-og-ernaring/vegetarisk-kosthold/infants-0-1-years/.

[63] Redecilla Ferreiro S., Moráis López A., Moreno Villares J.M., Leis Trabazo R., José Díaz J., Sáenz de Pipaón M., Blesa L., Campoy C., Ángel Sanjosé M., Gil Campos M. (2019). Position paper on vegetarian diets in infants and children. Committee on Nutrition and Breastfeeding of the Spanish Paediatric Association. An. Pediatría.

[64] Klapp A.L., Feil N., Risius A. (2022). A Global Analysis of National Dietary Guidelines on Plant-Based Diets and Substitutions for Animal-Based Foods. Curr. Dev. Nutr..

[65] Recenzijska Skupina Strateškega Sveta za Prehrano Strateški Svet za Prehrano o Recenziji Smernic za Prehranjevanje v Vzgojno-izobraževalnih Zavodih|GOV.SI. https://www.gov.si/novice/2023-04-04-strateski-svet-za-prehrano-o-recenziji-smernic-za-prehranjevanje-v-vzgojno-izobrazevalnih-zavodih/.

[66] Extended Professional College for Paediatrics of Slovenia 8 Korespodenčna Seja v Letu 2022. https://www.gov.si/assets/ministrstva/MZ/RSK-za-pediatrijo-9.-korespondencna-seja-23.12.2022.pdf.

[67] Ministry of Health of Slovenia The Possibility of a Vegan Meal in Educational Institutions. https://predlagam.vladi.si/predlog/10115.

[68] Orel R., Sedmak M., Fidler Mis N. (2014). Vegetarijanska prehrana pri otrocih—Praktična navodila. Zdrav. Vestn..

[69] Agostoni C., Decsi T., Fewtrell M., Goulet O., Kolacek S., Koletzko B., Michaelsen K.F., Moreno L., Puntis J., Rigo J. (2008). Complementary feeding: A commentary by the ESPGHAN Committee on Nutrition. J. Pediatr. Gastroenterol. Nutr..

[70] Choi Y., Larson N., Steffen L.M., Schreiner P.J., Gallaher D.D., Duprez D.A., Shikany J.M., Rana J.S., Jacobs D.R. (2021). Plant-centered diet and risk of incident cardiovascular disease during young to middle adulthood. J. Am. Heart Assoc..

[71] Dietitians of Canada Eating Guidelines for Vegans. https://www.mountsinai.on.ca/care/fammed/patient-resources/nutrition/vegan.pdf.

[72] Bratanič B., Fidler Mis N., Hlastan Ribič C., Poličnik R., Širca Čampa A., Kosem R., Fajdiga Tur V., Vertnik L., Povhe Jemec K. (2010). Healthy Eating Guidelines for Babies.

[73] Simčič I., Poličnik R., Gregorič M., Pograjc L., Kljajič Garbajs L., Kresal Sterniša B., Zobec U. Guideline for Nutrition in Educational Institutions. https://www.gov.si/assets/ministrstva/MIZS/Dokumenti/Osnovna-sola/Smernice_prehrana_2010.pdf.

[74] National Institute of Public Health of Slovenia Expert Opinion on Vegetarianism and China Studies. https://www.nijz.si/sl/strokovno-mnenje-glede-vegetarijanstva-in-kitajske-studije.

[75] Dietitians of Canada Healthy Eating Guidelines for Vegans. https://dietitianonadietdotcom.files.wordpress.com/2017/11/healthy-eating-for-vegans.pdf.

[76] Davis B., Melina V. (2014). Becoming Vegan: The Complete Guide to Adopting a Healthy Plant-Based Diet.

[77] Richter M., Boeing H., Grünewald-Funk D., Heseker H., Kroke A., Leschik-Bonnet E., Oberritter H., Strohm D., Watzl B. (2016). Vegan Diet. Ernaehrungs Umsch..

[78] Canadian Paediatric Society Vegetarian Diets for Children and Teens. https://caringforkids.cps.ca/handouts/healthy-living/vegetarian_diets_for_children_and_teens.

[79] Italian Society of Preventive and Social Pediatricians, Italian Federation of Medicine, Italian Federation of Adolescent Medicine, Italian Society of Perinatal Diete Vegetariane in Gravidanza ed in Età Evolutiva. https://www.sipps.it/wp/wp-content/uploads/2020/04/Position-Paper-Diete-vegetariane-2017.pdf.

[80] Finnish Food Authority Vegan Diet. https://www.ruokavirasto.fi/en/foodstuffs/healthy-diet/nutrition-and-food-recommendations/vegan-diet/.

[81] Service B.N.H. The Vegan Diet. https://www.nhs.uk/live-well/eat-well/how-to-eat-a-balanced-diet/the-vegan-diet/.

[82] Dietitians of Australia What Is a Vegetarian Diet?. https://dietitiansaustralia.org.au/health-advice/vegetarian-diet.

[83] Physicians Committee for Responsible Medicine A Vegan Diet During Pregnancy. https://www.pcrm.org/good-nutrition/plant-based-diets/pregnancy.

[84] Permanente K. Vegan Diet. https://healthy.kaiserpermanente.org/colorado/health-wellness/health-encyclopedia/he.vegan-diet.abq2485.

[85] Světnička M., Heniková M., Selinger E., Ouřadová A., Potočková J., Kuhn T., Gojda J., El-Lababidi E. (2023). Prevalence of iodine deficiency among vegan compared to vegetarian and omnivore children in the Czech Republic: Cross-sectional study. Eur. J. Clin. Nutr..

[86] Světnička M., Sigal A., Selinger E., Heniková M., El-lababidi E., Gojda J. (2022). Cross-Sectional Study of the Prevalence of Cobalamin Deficiency and Vitamin B12 Supplementation Habits among Vegetarian and Vegan Children in the Czech Republic. Nutrients.

[87] Elliott L.J., Keown-Stoneman C.D.G., Birken C.S., Jenkins D.J.A., Borkhoff C.M., Maguire J.L. (2022). Vegetarian Diet, Growth, and Nutrition in Early Childhood: A Longitudinal Cohort Study. Pediatrics.

[88] Hebbelinck M., Clarys P., De Malsche A. (1999). Growth, development, and physical fitness of Flemish vegetarian children, adolescents, and young adults. Am. J. Clin. Nutr..

[89] Nathan I., Hackett A.F., Kirby S. (1997). A longitudinal study of the growth of matched pairs of vegetarian and omnivorous children, aged 7-11 years, in the north-west of England. Eur. J. Clin. Nutr..

[90] Henjum S., Groufh-Jacobsen S., Aakre I., Folven Gjendedal E.L., Marthinsen Langfjord M., Heen E., Sele V., Andersson M. (2023). Thyroid function and urinary concentrations of iodine, selenium, and arsenic in vegans, lacto-ovo vegetarians and pescatarians. Eur. J. Nutr..

[91] Mendes D., Alves C., Silverio N., Marques F.B. (2019). Prevalence of Undiagnosed Hypothyroidism in Europe: A Systematic Review and Meta-Analysis. Eur. Thyroid J..

[92] Biondi B., Cappola A.R., Cooper D.S. (2019). Subclinical Hypothyroidism: A Review. JAMA.

[93] Tonstad S., Nathan E., Oda K., Fraser G. (2013). Vegan Diets and Hypothyroidism. Nutrients.

[94] Nicol K., Nugent A.P., Woodside J.V., Hart K.H., Bath S.C. (2023). Iodine and plant-based diets—A narrative review and calculation of iodine content. Br. J. Nutr..

[95] Biban B.G., Lichiardopol C. (2017). Iodine Deficiency, Still a Global Problem?. Curr. Health Sci. J..

[96] Hatch-McChesney A., Lieberman H.R. (2022). Iodine and Iodine Deficiency: A Comprehensive Review of a Re-Emerging Issue. Nutrients.

[97] Hovinen T., Korkalo L., Freese R., Skaffari E., Isohanni P., Niemi M., Nevalainen J., Gylling H., Zamboni N., Erkkola M. (2021). Vegan diet in young children remodels metabolism and challenges the statuses of essential nutrients. EMBO Mol. Med..

[98] Desmond M.A., Sobiecki J.G., Jaworski M., Płudowski P., Antoniewicz J., Shirley M.K., Eaton S., Ksiązyk J., Cortina-Borja M., De Stavola B. (2021). Growth, body composition, and cardiovascular and nutritional risk of 5- to 10-y-old children consuming vegetarian, vegan, or omnivore diets. Am. J. Clin. Nutr..

[99] Morency M.E., Birken C.S., Lebovic G., Chen Y., L’Abbé M., Lee G.J., Maguire J.L. (2017). Association between noncow milk beverage consumption and childhood height. Am. J. Clin. Nutr..

[100] Qin L.Q., He K., Xu J.Y. (2009). Milk consumption and circulating insulin-like growth factor-I level: A systematic literature review. Int. J. Food Sci. Nutr..

[101] Green J., Cairns B.J., Casabonne D., Wright F.L., Reeves G., Beral V. (2011). Height and cancer incidence in the Million Women Study: Prospective cohort, and meta-analysis of prospective studies of height and total cancer risk. Lancet Oncol..

[102] Krieg S., Roderburg C., Krieg A., Luedde T., Loosen S.H., Kostev K. (2022). The association between body height and cancer: A retrospective analysis of 784,192 outpatients in Germany. J. Cancer Res. Clin. Oncol..

[103] Gremke N., Griewing S., Kalder M., Kostev K. (2022). Positive association between body height and breast cancer prevalence: A retrospective study with 135,741 women in Germany. Breast Cancer Res. Treat..

[104] Zhou E., Wang L., Santiago C.N., Nanavati J., Rifkin S., Spence E., Hylind L.M., Gills J.J., Luna L.L., Kafonek D.R. (2022). Adult-Attained Height and Colorectal Cancer Risk: A Cohort Study, Systematic Review, and Meta-Analysis. Cancer Epidemiol. Biomark. Prev..

[105] Willett W.C., Ludwig D.S. (2020). Milk and Health. N. Engl. J. Med..

[106] Rodríguez-Borjabad C., Narveud I., Christensen J.J., Ulven S.M., Malo A.I., Ibarretxe D., Girona J., Torvik K., Bogsrud M.P., Retterstøl K. (2021). Dietary intake and lipid levels in Norwegian and Spanish children with familial hypercholesterolemia. Nutr. Metab. Cardiovasc. Dis..

[107] Weder S., Hoffmann M., Becker K., Alexy U., Keller M. (2019). Energy, macronutrient intake, and anthropometrics of vegetarian, vegan, and omnivorous children (1–3 years) in Germany (VeChi diet study). Nutrients.

[108] Weder S., Zerback E.H., Wagener S.M., Koeder C., Fischer M., Alexy U., Keller M. (2022). How Does Selenium Intake Differ among Children (1–3 Years) on Vegetarian, Vegan, and Omnivorous Diets? Results of the VeChi Diet Study. Nutrients.

[109] Alexy U., Fischer M., Weder S., Längler A., Michalsen A., Sputtek A., Keller M. (2021). Nutrient intake and status of german children and adolescents consuming vegetarian, vegan or omnivore diets: Results of the vechi youth study. Nutrients.

[110] Hohoff E., Zahn H., Weder S., Fischer M., Längler A., Michalsen A., Keller M., Alexy U. (2022). Food Costs of Children and Adolescents Consuming Vegetarian, Vegan or Omnivore Diets: Results of the Cross-Sectional VeChi Youth Study. Nutrients.

[111] Bailey A.D.L., Fulgoni V.L., Shah N., Patterson A.C., Gutierrez-Orozco F., Mathews R.S., Walsh K.R. (2021). Nutrient Intake Adequacy from Food and Beverage Intake of US Children Aged 1–6 Years from NHANES 2001–2016. Nutrients.

[112] Najjar R.S., Gewirtz A.T. (2023). Plant-Based Diets: A Path to Ending CVD as We Know It?. Nutrients.

[113] Chen G., Su M., Chu X., Wei Y., Chen S., Zhou Y., Liu Z., Zhang Z. (2022). Plant-based diets and body composition in Chinese omnivorous children aged 6–9 years old: A cross-sectional study. Front. Nutr..

[114] Macknin M., Kong T., Weier A., Worley S., Tang A.S., Alkhouri N., Golubic M. (2015). Plant-Based, No-Added-Fat or American Heart Association Diets: Impact on Cardiovascular Risk in Obese Children with Hypercholesterolemia and Their Parents. J. Pediatr..

[115] Macknin M., Stegmeier N., Thomas A., Worley S., Li L., Hazen S.L., Tang W.H.W. (2021). Three Healthy Eating Patterns and Cardiovascular Disease Risk Markers in 9 to 18 Year Olds with Body Mass Index >95%: A Randomized Trial. Clin. Pediatr..

[116] Seljak B.K., Valenčič E., Hristov H., Hribar M., Lavriša Ž., Kušar A., Žmitek K., Krušič S., Gregorič M., Blaznik U. (2021). Inadequate Intake of Dietary Fibre in Adolescents, Adults, and Elderlies: Results of Slovenian Representative SI. Menu Study. Nutrients.

[117] Pravst I., Lavriša Ž., Hribar M., Hristov H., Kvarantan N., Seljak B.K., Gregorič M., Blaznik U., Gregorič N., Zaletel K. (2021). Dietary Intake of Folate and Assessment of the Folate Deficiency Prevalence in Slovenia Using Serum Biomarkers. Nutrients.

[118] Hribar M., Hristov H., Lavriša Ž., Seljak B.K., Gregorič M., Blaznik U., Žmitek K., Pravst I. (2021). Vitamin D Intake in Slovenian Adolescents, Adults, and the Elderly Population. Nutrients.

[119] Lavriša Ž., Hristov H., Hribar M., Koroušić Seljak B., Gregorič M., Blaznik U., Zaletel K., Oblak A., Osredkar J., Kušar A. (2022). Dietary Iron Intake and Biomarkers of Iron Status in Slovenian Population: Results of SI.Menu/Nutrihealth Study. Nutrients.

[120] Lavriša Ž., Hristov H., Hribar M., Žmitek K., Kušar A., Koroušić Seljak B., Gregorič M., Blaznik U., Gregorič N., Zaletel K. (2022). Dietary Intake and Status of Vitamin B12 in Slovenian Population. Nutrients.

[121] Fidler Mis N., Kobe H., Štimec M. (2012). Dietary intake of macro-and micronutrients in Slovenian adolescents: Comparison with reference values. Ann. Nutr. Metab..

[122] Štimec M., Kobe H., Smole K., Kotnik P., Širca-Čampa A., Zupančič M., Battelino T., Kržišnik C., Fidler Mis N. (2009). Adequate iodine intake of Slovenian adolescents is primarily attributed to excessive salt intake. Nutr. Res..

[123] Zdešar Kotnik K., Jurak G., Gregorič M., Koroušić Seljak B., Golja P., Petelin A., Plevnik M., Urška Č., Žvanut B., Pucer P., Bulič M. (2019). Evaluation of nutrition among adolescents in Slovenia. Health of Children and Adolescents.

[124] Zdešar Kotnik K., Jurak G., Starc G., Golja P. (2017). Faster, Stronger, Healthier: Adolescent-Stated Reasons for Dietary Supplementation. J. Nutr. Educ. Behav..

[125] Sorić M., Jurak G., Đurić S., Kovač M., Strel J., Starc G. (2020). Increasing trends in childhood overweight have mostly reversed: 30 years of continuous surveillance of Slovenian youth. Sci. Rep..

[126] Radulović A., Jurak G., Leskošek B., Starc G., Blagus R. (2022). Secular trends in physical fitness of Slovenian boys and girls aged 7 to 15 years from 1989 to 2019: A population-based study. Sci. Rep..

[127] Neufingerl N., Eilander A. (2023). Nutrient Intake and Status in Children and Adolescents Consuming Plant-Based Diets Compared to Meat-Eaters: A Systematic Review. Nutrients.

[128] O’connell J.M., Dibley M.J., Bs M.B., Sierra J., Wallace B., Marks J.S., Yip R. (1989). Growth of Vegetarian Children: The Farm Study. Pediatrics.

[129] Welch A.A., Shakya-Shrestha S., Lentjes M.A.H., Wareham N.J., Khaw K.T. (2010). Dietary intake and status of n-3 polyunsaturated fatty acids in a population of fish-eating and non-fish-eating meat-eaters, vegetarians, and vegans and the precursor-product ratio of α-linolenic acid to long-chain n-3 polyunsaturated fatty acids: Results from the EPIC-Norfolk cohort. Am. J. Clin. Nutr..

[130] van der Wurff I.S.M., Meyer B.J., de Groot R.H.M. (2020). Effect of Omega-3 Long Chain Polyunsaturated Fatty Acids (n-3 LCPUFA) Supplementation on Cognition in Children and Adolescents: A Systematic Literature Review with a Focus on n-3 LCPUFA Blood Values and Dose of DHA and EPA. Nutrients.

[131] Arellanes I.C., Choe N., Solomon V., He X., Kavin B., Martinez A.E., Kono N., Buennagel D.P., Hazra N., Kim G. (2020). Brain delivery of supplemental docosahexaenoic acid (DHA): A randomized placebo-controlled clinical trial. EBioMedicine.

[132] Sarter B., Kelsey K.S., Schwartz T.A., Harris W.S. (2015). Blood docosahexaenoic acid and eicosapentaenoic acid in vegans: Associations with age and gender and effects of an algal-derived omega-3 fatty acid supplement. Clin. Nutr..

[133] Sherzai A.Z., Sherzai A.N., Sherzai D. (2022). A Systematic Review of Omega-3 Consumption and Neuroprotective Cognitive Outcomes. Am. J. Lifestyle Med..

[134] Harris W.S., Tintle N.L., Imamura F., Qian F., Korat A.V.A., Marklund M., Djoussé L., Bassett J.K., Carmichael P.H., Chen Y.Y. (2021). Blood n-3 fatty acid levels and total and cause-specific mortality from 17 prospective studies. Nat. Commun..

[135] Kobe H., Štimec M., Ribič C.H., Fidler Mis N., Ribic C.H., Mis F. (2012). Food intake in Slovenian adolescents and adherence to the Optimized Mixed Diet: A nationally representative study. Public Health Nutr..

[136] Burns J.L., Bhattacharjee A., Darlington G., Haines J., Ma D.W.L. (2023). The Guelph Family Health Study Parental Cooking Confidence is Associated with Children’s Intake of Fish and Seafood. Can. J. Diet. Pract. Res..

[137] Kranz S., Jones N.R.V., Monsivais P. (2017). Intake Levels of Fish in the UK Paediatric Population. Nutrients.

[138] Koletzko B., Uauy R., Palou A., Kok F., Hornstra G., Eilander A., Moretti D., Osendarp S., Zock P., Innis S. (2010). Dietary intake of eicosapentaenoic acid (EPA) and docosahexaenoic acid (DHA) in children—A workshop report. Br. J. Nutr..

[139] Willett W., Rockström J., Loken B., Springmann M., Lang T., Vermeulen S., Garnett T., Tilman D., DeClerck F., Wood A. (2019). Food in the Anthropocene: The EAT–Lancet Commission on healthy diets from sustainable food systems. Lancet.

[140] Seth D., Poowutikul P., Pansare M., Kamat D. (2020). Food Allergy: A Review. Pediatr. Ann..

[141] Arya C., Jantwal C. (2017). A Review on Identified Major Food Allergens: Characteristics and Role in Food Allergy. Indian J. Nutr. Diet..

[142] Reese I., Schäfer C., Ballmer-Weber B., Beyer K., Dölle-Bierke S., van Dullemen S., Jappe U., Müller S., Schnadt S., Treudler R. (2023). Vegan diets from an allergy point of view—Position paper of the DGAKI working group on food allergy. Allergol. Sel..

[143] Kahleova H., Sutton M., Maracine C., Nichols D., Monsivais P., Holubkov R., Barnard N.D. (2023). Vegan Diet and Food Costs Among Adults With Overweight: A Secondary Analysis of a Randomized Clinical Trial. JAMA Netw. Open.

[144] Pais D.F., Marques A.C., Fuinhas J.A. (2022). The cost of healthier and more sustainable food choices: Do plant-based consumers spend more on food?. Agric. Food Econ..

[145] Umeå University Vegan Diet in Small Children (VHS). https://clinicaltrials.gov/study/NCT05442281.

[146] Sabaté J., Wien M. (2010). Vegetarian diets and childhood obesity prevention. Am. J. Clin. Nutr..

[147] Tran E., Dale H.F., Jensen C., Lied G.A. (2020). Effects of Plant-Based Diets on Weight Status: A Systematic Review. Diabetes Metab. Syndr. Obes. Targets Ther..

